# Automated HER2 Scoring in Breast Cancer Images Using Deep Learning and Pyramid Sampling

**DOI:** 10.34133/bmef.0048

**Published:** 2024-07-23

**Authors:** Sahan Yoruc Selcuk, Xilin Yang, Bijie Bai, Yijie Zhang, Yuzhu Li, Musa Aydin, Aras Firat Unal, Aditya Gomatam, Zhen Guo, Darrow Morgan Angus, Goren Kolodney, Karine Atlan, Tal Keidar Haran, Nir Pillar, Aydogan Ozcan

**Affiliations:** ^1^ Electrical and Computer Engineering Department, University of California, Los Angeles, Los Angeles, CA, USA.; ^2^ Bioengineering Department, University of California, Los Angeles, Los Angeles, CA, USA.; ^3^ California NanoSystems Institute, University of California, Los Angeles, Los Angeles, CA, USA.; ^4^ Department of Pathology and Laboratory Medicine, University of California at Davis, Sacramento, CA, USA.; ^5^ Bnai-Zion Medical Center, Haifa, Israel.; ^6^ Hadassah Hebrew University Medical Center, Jerusalem, Israel.; ^7^ David Geffen School of Medicine, University of California, Los Angeles, Los Angeles, CA, USA.

## Abstract

**Objective and Impact Statement:** Human epidermal growth factor receptor 2 (HER2) is a critical protein in cancer cell growth that signifies the aggressiveness of breast cancer (BC) and helps predict its prognosis. Here, we introduce a deep learning-based approach utilizing pyramid sampling for the automated classification of HER2 status in immunohistochemically (IHC) stained BC tissue images. **Introduction:** Accurate assessment of IHC-stained tissue slides for HER2 expression levels is essential for both treatment guidance and understanding of cancer mechanisms. Nevertheless, the traditional workflow of manual examination by board-certified pathologists encounters challenges, including inter- and intra-observer inconsistency and extended turnaround times. **Methods:** Our deep learning-based method analyzes morphological features at various spatial scales, efficiently managing the computational load and facilitating a detailed examination of cellular and larger-scale tissue-level details. **Results:** This approach addresses the tissue heterogeneity of HER2 expression by providing a comprehensive view, leading to a blind testing classification accuracy of 84.70%, on a dataset of 523 core images from tissue microarrays. **Conclusion:** This automated system, proving reliable as an adjunct pathology tool, has the potential to enhance diagnostic precision and evaluation speed, and might substantially impact cancer treatment planning.

## Introduction

Breast cancer (BC) is one of the most common types of cancer globally, ranking as the most prevalent cancer among women (excluding nonmelanoma skin cancers) and the second-leading cause of cancer-related deaths among women after lung cancer [1,2]. The complex and varied nature of BC necessitates accurate histological diagnostic procedures, such as determining the status of the human epidermal growth factor receptor 2 (HER2) [3]. HER2 protein plays an important role in the growth of cancer cells and is a key indicator of BC aggressiveness. The level of HER2 protein expression has prognostic and predictive value in determining patient outcomes [4].

**Fig. 1.**
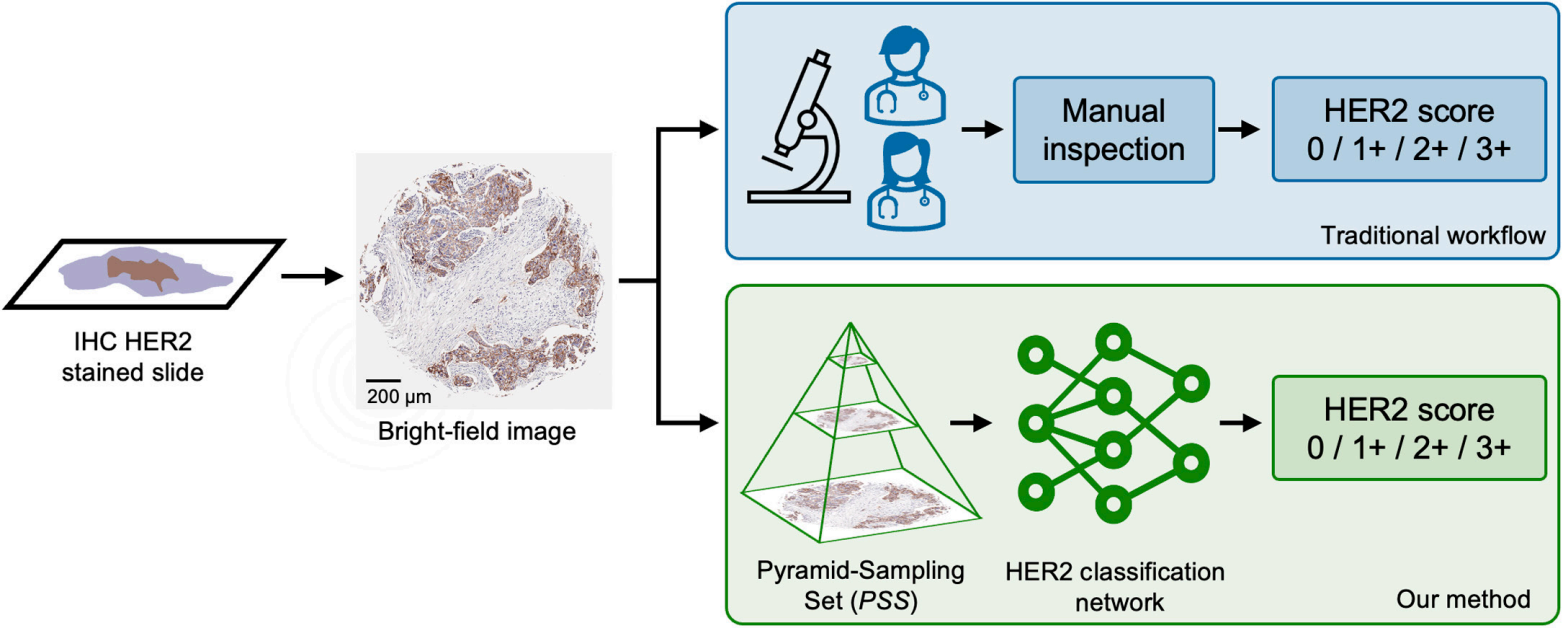
Comparison of traditional HER2 scoring and the presented DL-based method. The traditional HER2 score evaluation depends on manual inspection of tissue slides by pathologists. Our presented methodology introduces automated HER2 scoring using pyramid sampling and a classification neural network.

In clinical practice, the assessment of HER2 status is mostly conducted through immunohistochemical (IHC) staining [5], followed by manual inspection of tissue slides by certified pathologists, a process depicted in Fig. 1. The American Society of Clinical Oncology/College of American Pathologists (ASCO/CAP) guidelines, published in 2018 [6] and affirmed in 2023 [7], outline specific scoring criteria for this assessment. A tumor is considered HER2 positive if it shows strong, complete, and intense membrane staining (3+) in more than 10% of tumor cells. If the staining is weak to moderate and complete in more than 10% of tumor cells, the case is scored as equivocal (2+). Cases with no staining or incomplete, barely perceptible membrane staining in 10% of tumor cells or less are classified as HER2 negative (0+ or 1+).

Although this traditional method is widely adopted, several challenges emerge in the manual evaluation of IHC slides. Reproducibility and concordance among pathologists are poor, and this may compromise diagnostic accuracy [8,9]. Additionally, this manual evaluation process is notably time-consuming and requires careful examination by pathologists. These challenges are further exacerbated in resource-constrained areas, where the availability of expert breast pathologists may be limited, making HER2 assessment even more challenging [10,11].

Therefore, there is a growing need for automated tools that can assist pathologists in the evaluation of IHC images [12] to streamline the assessment of HER2 status, enhancing its efficiency, consistency, and reliability [13,14]. Such tools need to reduce the time required for analysis, decrease false positives and negatives, and reduce variability in measurements and require rigorous test sets for evaluation [15]. However, due to the lack of high-quality large public datasets with rigorous established labels, most of the demonstrated approaches are only assessed on small, hand-picked image patches, which failed to capture the tissue heterogeneity and sample variations typically encountered in clinical settings. A notable contest [16] entailed 86 whole-slide images (WSIs) where only 28 were selected for testing. Strong discordance between experts and clinical reports was observed with most discrepancy falling between HER2 1+ and 2+. Nonetheless, automated HER2 scoring systems have seen considerable advances through various computational methodologies. Early research in this domain utilized standard image processing techniques and machine learning methods. Further work investigated the use of local binary patterns (LBPs) and color features in conjunction with machine learning algorithms. Leveraging intensity and color features alongside uniform LBP, Singh and Mukundan [17] achieved a 91.1% accuracy using a neural network classifier on a filtered set of 371 image patches, specifically excluding outliers and ensuring a minimum of 80% content of interest within each patch; successive research by the same group deployed characteristic curves and uniform LBP features with logistic regression and support vector machine (SVM) classifiers for HER2 score assessment while omitting image tiles with less than 40% of the region of interest (ROI) [18].

The adoption of deep neural networks (DNNs), especially convolutional neural networks (CNNs), marks a recent shift in computational pathology, which is largely driven by neural networks’ ability to analyze and interpret complex patterns in histopathology images [19]. For instance, CNNs processing 128 × 128-pixel HER2 image patches for score classification achieved a 97.7% accuracy on 119 core regions from 81 WSIs by manually selecting small, reliable regions for classification [20]. Combining SVM, random forest, and a CNN for HER2 scoring with color deconvolution and watershed segmentation resulted in an accuracy of 83% [21]. Additionally, fully connected long short-term memory (LSTM) networks have been used for segmenting and labeling cell membranes and nuclei in HER2-stained tissue samples, reaching 98.33% accuracy on a set of 752 image patches from 79 WSIs, highlighting a selective analysis with data exclusion [22]. Other approaches, including deep reinforcement learning for ROI-based score prediction, and a modified U-Net architecture for WSI segmentation and tissue classification, have demonstrated HER2 classification accuracies of 79.4% and 87% over datasets of 86 and 127 WSIs, respectively [23,24]. In addition to these, there has been increasing interest in exploring multiple instance learning (MIL) methods to enhance the analysis of histopathological images. MIL is a form of weakly supervised learning where training instances are arranged in sets, called bags, and a label is provided for the entire bag—usually on WSI level—instead of individual instances/image patches [25]. Liu et al*.* [26] implemented a MIL-based weakly supervised learning framework, evaluated on a dataset of 251 slides and achieving a 55% accuracy in classifying HER2.

The preceding studies have delved into automating HER2 scoring employing diverse techniques, ranging from image processing to advanced machine learning techniques, predominantly using patches from a single-resolution level, neglecting features observable at the broader tissue context, which is essential for precise HER2 evaluation. Additionally, investigations employing MIL necessitate an exhaustive analysis of every potential high-resolution patch within WSIs, resulting in a heavy computational load. Furthermore, most of the existing methods preselect small ROIs from WSIs or tissue cores in their training and testing. Such sampling strategies underrepresent tissue complexity and variability, potentially leading to overestimated performance metrics and a lack of generalizability.

**Fig. 2.**
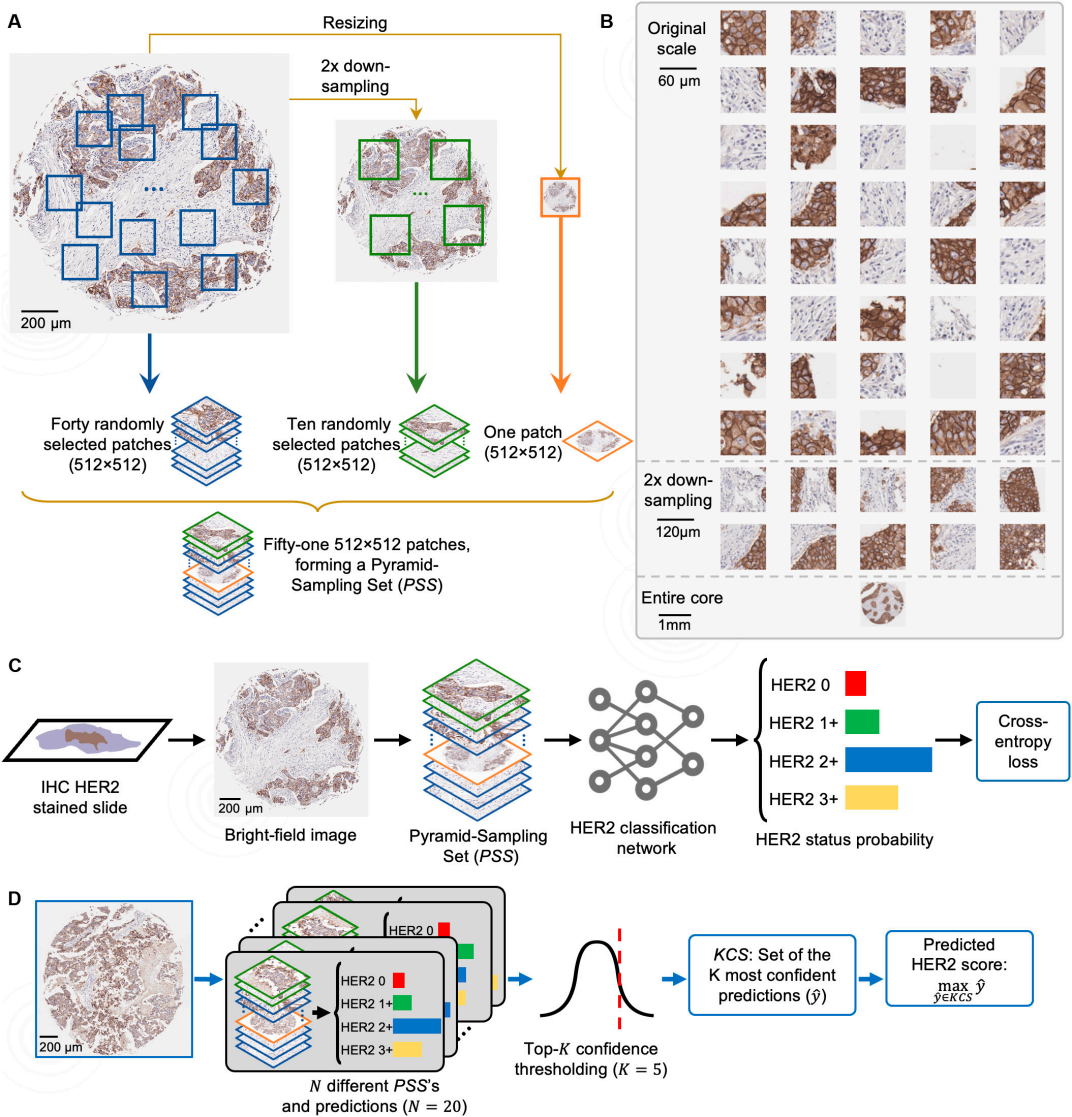
Overview of the automated HER2 score classification framework. (A) Formation of a *PSS*, detailing the extraction of multi-resolution patches from tissue images. (B) Instance of a *PSS*, showing 40 randomly selected patches from the original resolution, 10 randomly selected patches from the half-resolution case (2×-downsampled), and the entire core image resized to the patch resolution. (C) Training process of the presented methodology involving pyramid sampling and backpropagation through a deep network. (D) Presented inference protocol, entailing the formation of multiple *PSS*s and the final HER2 score prediction as the maximum HER2 score among the predictions with top-*k* confidence.

In this work, we introduce an automated, deep learning (DL)-based HER2 score classification framework, illustrated in Fig. 1. Contrasting the aforementioned approaches, our method is based on a pyramid sampling strategy and a HER2 score inference protocol (as shown in Fig. 2A and D), addressing the classification challenge of HER2 expression heterogeneity. By using a randomly selected subset of high-resolution patches rather than exhaustively analyzing all possible ones, we greatly enhance computational efficiency without compromising HER2 score inference accuracy. Moving beyond conventional single-resolution-based image analyses, our pyramid sampling framework integrates detailed cellular features with broader tissue architecture, offering a comprehensive representation of HER2 expression patterns and a complete perspective on tissue heterogeneity. Our inference protocol analyzes morphological features at multiple spatial scales, efficiently balancing detailed cellular analysis with broader tissue examination. This approach, which captures and integrates patches of varying scales from high-resolution images into a Pyramid-Sampling Set (*PSS*) for DL-based evaluation, not only tackles the issue of HER2 expression heterogeneity but also achieves an accuracy of 84.70% in blind testing across 523 tissue core images obtained from 300 patients. This automated approach can help standardize HER2 assessment, streamline pathologists’ workflow, and improve diagnostic accuracy.

## Results

Our study introduces a DL-based HER2 classification method utilizing *PSS*s to analyze BC tissue samples. The classification network was trained on a dataset of 1,462 core images from 823 patients, with an additional set of 162 cores from 149 patients used for validation. The efficacy of the model was blindly evaluated using a set of 523 core images from 300 patients that were not previously seen by the model during the training or validation phases. We evaluated the performance of our model with both qualitative and quantitative analyses. While creating the ground truth of our dataset, we involved 5 board-certified pathologists to independently score the cores. This approach mitigated the risk of relying on possibly inaccurate patient records due to tissue heterogeneity, providing a robust methodology for dataset labeling that prioritizes precision in reflecting real-world diagnostic scenarios. We introduced a voting protocol (detailed in the Methods section) to resolve interpathologist variability and to achieve consensus in core evaluations. Nondiagnostic cores were excluded to ensure the high quality and diagnostic relevance of the compiled dataset. Overall, we compiled a comprehensive and accurately labeled dataset of 2,147 cores from 1,272 patients.

Our model’s training and testing protocols are detailed in Fig. 2C and D. During training, each core image is transformed into a *PSS* and fed into the DL model with its ground truth label. We compute a cross-entropy loss to optimize the model, which, upon convergence, enters the testing phase. In testing, for each core image, *N* independent *PSS*s are generated. We then conduct a forward process on these sets, selecting the top *k* predictions with the highest confidence, forming the *k*-Confident Selection Set (*KCS*). The final score is the highest score within the *KCS*, effectively addressing the heterogeneity of HER2 expression. Therefore, the 2 key hyperparameters in this process are *N*, the number of independent *PSS*s generated per tissue core, and *k*, the number of high-confidence predictions used for final scoring. This method ensures a diverse representation of HER2 status and focuses on the most confident predictions to enhance HER2 scoring accuracy. By prioritizing predictions with the highest confidence, our approach reduces the chance of inaccuracies due to lower-confidence inferences, ensuring that the final HER2 score is based on significant expressions. This methodology not only improves the reliability of HER2 scoring but also captures essential expressions critical for a comprehensive assessment of HER2 status in breast tissue sections.

**Fig. 3.**
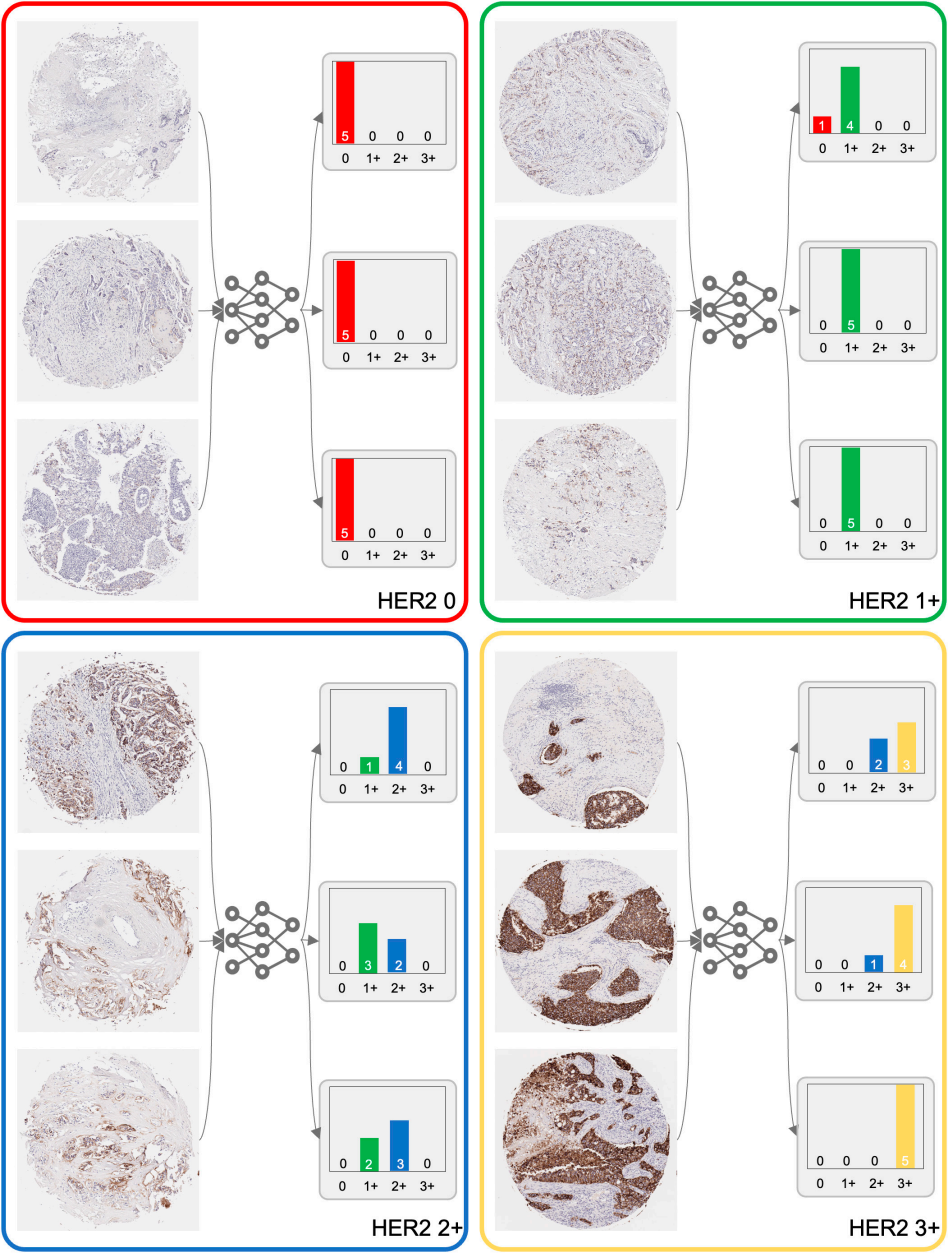
Distribution of the predicted HER2 scores for 12 randomly selected test samples, as determined by the *PSS* approach. For each sample, *N* = 20 independent *PSS* predictions are generated, and the histograms display the HER2 score distributions from the *k* = 5 *PSS*s with the highest confidence levels. The final HER2 score prediction for each sample is generated by the maximum score from these top 5 confidence *PSS*s. Samples are grouped and color-coded according to their consensus HER2 score categories: 0, 1+, 2+, and 3+.

We demonstrate the capability of our automated HER2 scoring system with 12 examples in Fig. 3. This figure illustrates the distribution of the predicted HER2 scores coming from the most confident 5 predictions for a subset of the test samples, which were accurately classified into the 4 HER2 categories. Each sample underwent evaluation by generating 20 independent *PSS* predictions, with the subsequent histograms depicting the score distributions from the 5 highest-confidence *PSS*s. These predictions were then grouped into color-coded categories corresponding to the consensus HER2 score: 0, 1+, 2+, and 3+.

A key observation from Fig. 3 is the influence of HER2 expression heterogeneity on the predictions of the *PSS*s with the highest confidence. For example, within the yellow-coded box highlighting HER2 3+ samples, there is a noticeable variation in the intensity of HER2 biomarker expression. The last core image within this grouping shows a pronounced level of HER2 positivity, which is consistently recognized across all 5 high-confidence *PSS* predictions as 3+. In contrast, the first core image of the same category exhibits a lower intensity of HER2 expression, leading to a slight variance where 2 of 5 high-confidence *PSS*s predict a 2+ score. Similarly, the green and blue boxes corresponding to HER2 1+ and HER2 2+ categories, respectively, also demonstrate this trend. The model’s predictions reflect the level of HER2 expression, with the majority of high-confidence *PSS*s aligning with the consensus category in most samples. However, some *PSS*s indicate adjacent categories, suggesting a borderline expression level. The red box, delineating HER2 0 samples, is particularly noteworthy, as all high-confidence *PSS*s consistently predict a HER2 score of 0.

**Fig. 4.**
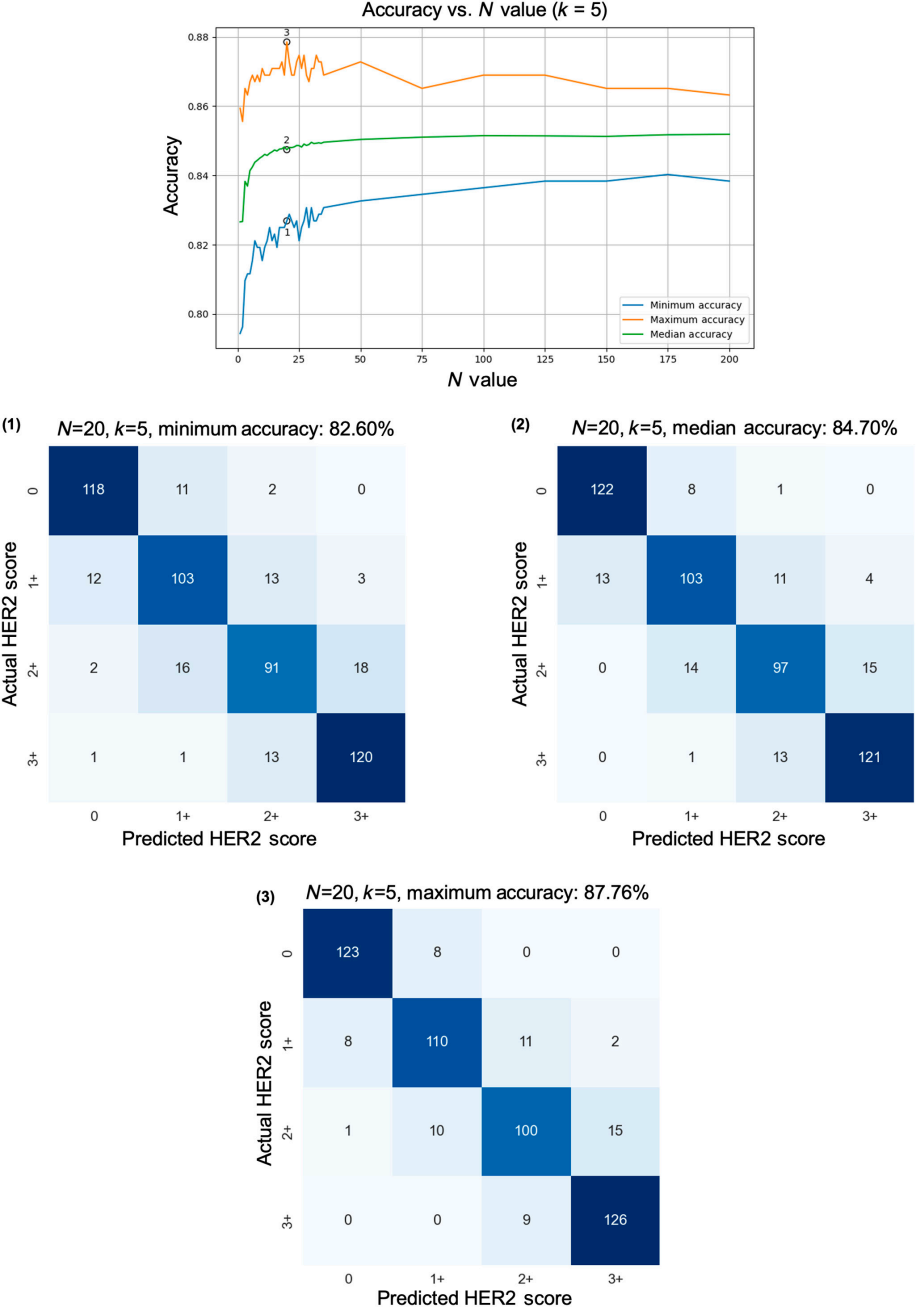
Relationship between the number of independent *PSS*s and HER2 scoring accuracy. The top plot displays how accuracy changes as a function of *N* used during inference, with a fixed confidence threshold parameter (*k* = 5). The accompanying confusion matrices exemplify the accuracy at distinct performance benchmarks—minimum, median, and maximum—achieved with *N* = 20 *PSS*s. These benchmarks were established through a Monte Carlo simulation designed to evaluate the influence of *PSS* selection randomness on the overall accuracy of the HER2 score classification system. Blind testing set includes 523 core images from 300 patients that were not previously seen by the model during the training or validation.

Monte Carlo simulations were also leveraged to reveal the effects of varying the number of independent *PSS*s. For each sample, we employed our converged model and tested samples with varying *N* and *k* values, as well as different sets of *PSS*s, to validate the stability and consistency of our model; refer to the Methods section for details. Classification accuracy was markedly improved as the number of *PSS*s increased up to a certain point, with a fixed confidence threshold parameter of *k* = 5 as shown in Fig. 4. A notable peak in accuracy is observed when *N* is set to 20, where the maximum classification accuracy reaches 87.76%. As we continued to increase *N* to 200, the accuracy gain became marginal. Confusion matrices corresponding to the minimum, median, and maximum accuracy benchmarks, achieved with *N* = 20 and *k* = 5, provide a quantitative view of the system’s performance as shown in Fig. 4. Specifically, the minimum accuracy recorded is 82.52%, the median accuracy stands at 84.70%, and the maximum accuracy reaches 87.76%. Initially, at lower values of *N*, there is a wider spread between these accuracy measures, indicating variability in the model’s performance. This implies that to decrease the variation in predictions caused by random sampling, employing a larger *N* is effective at the cost of testing speed. In doing so, the performance is expected to align with the median value observed under the default configuration (*N* = 20, *k* = 5).

**Fig. 5.**
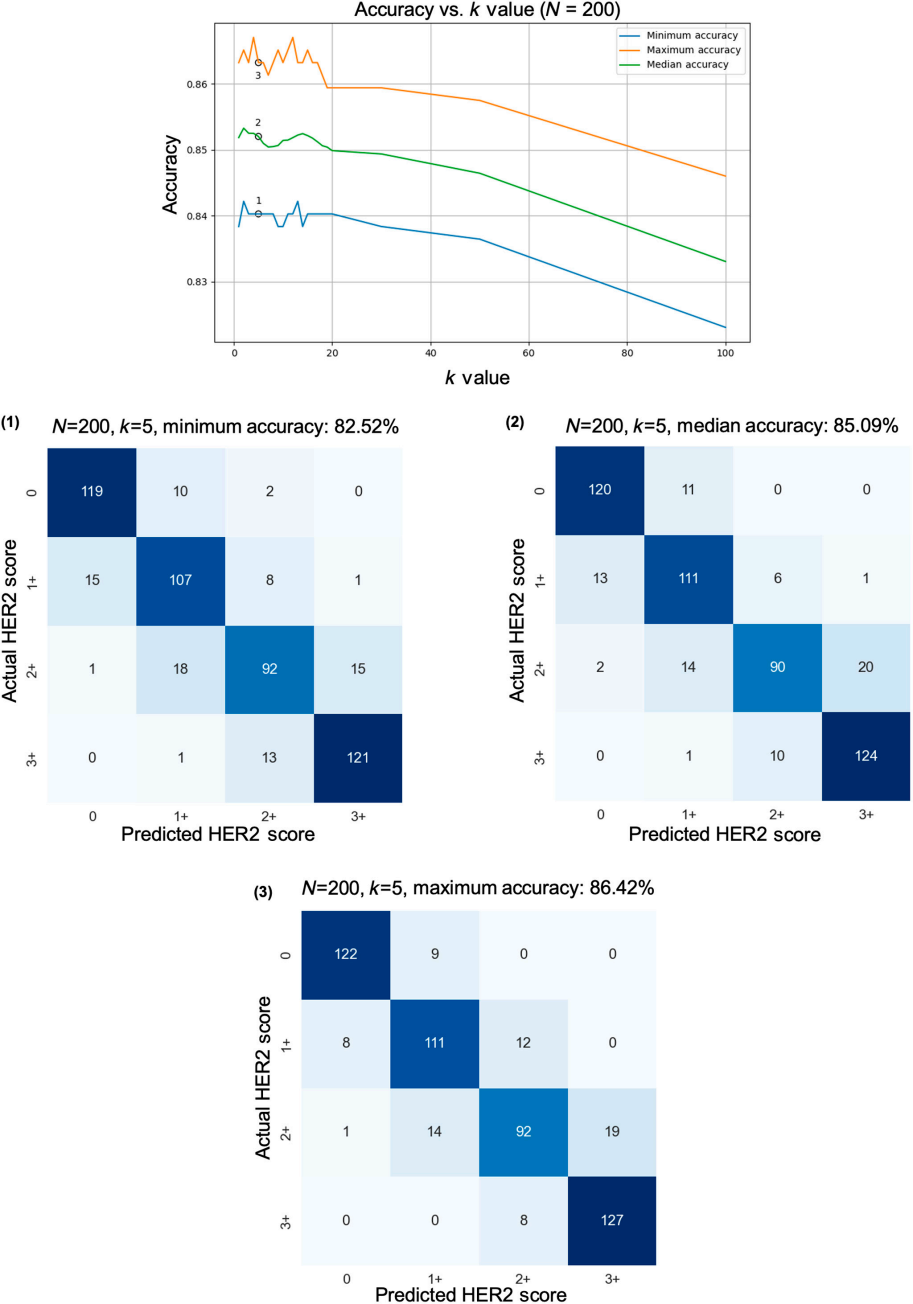
Analysis of HER2 scoring precision as a function of *k* with a fixed number of independent *PSS*s (*N* = 200). The graph traces accuracy variations across different *k* values, while the confusion matrices document the system’s performance at minimum, median, and maximum accuracy values for *k* = 5. These analyses examine the impact of the confidence threshold parameter (*k*) on the overall accuracy of HER2 score predictions. Blind testing set includes 523 core images from 300 patients that were not previously seen by the model during the training or validation.

Next, we focused on the precision of classification accuracy across varying confidence threshold parameters, denoted by the parameter *k*, while holding the number (*N*) of independent *PSS*s used in the inference protocol constant at 200, shown in Fig. 5. This figure highlights the delicate balance between the confidence in prediction and the precision of the final score, and it illustrates the performance metrics at different accuracy levels using confusion matrices. The accuracy trends depicted in the graph provide an illustration of the model’s classification performance against varying *k* values. Notably, when the confidence threshold parameter (*k*) value exceeded 20, there was a pronounced decrement in the model’s accuracy. This was consistently observed across the minimum, median, and maximum accuracy rates. This trend suggests that a *k* value within the range of 1 to 20 maintains optimal classification performance, whereas higher *k* values lead to a marked reduction in accuracy.

We also evaluated the class-based specificity to understand the model’s ability to correctly identify negatives for each class. The specificity values for each class were calculated as follows: HER2 0: 96.43%, HER2 1+: 92.09%, HER2 2+: 94.46%, and HER2 3+: 93.56%. Notably, the model achieved the highest specificity for class 0, suggesting a strong ability to correctly identify non-HER2-positive cases. The slightly lower specificity for class 1 indicates a minor increase in false positives, which may be due to the overlap in features between adjacent HER2 scores. Nevertheless, the high specificity values across all the classes demonstrate the model’s robust performance in distinguishing among different HER2 scores.

Additionally, we analyzed the receiver operating characteristic (ROC) curves and area under the curve (AUC) for the 3 binary classification tasks to further assess the model’s performance. The AUC values for these tasks were as follows: HER2 0 versus HER2 1+, 2+, and 3+: 0.9848, HER2 0 and 1+ versus HER2+ and 3+: 0.9645, and HER2 0, 1+, and 2+ versus class 3+: 0.8555. The high AUC value for HER2 0 versus HER2 1+, 2+, and 3+ further highlights the model’s discriminative ability in identifying non-HER2-positive cases. The AUC value for HER2 0 and 1+ versus HER2+ and 3+ indicates strong performance, with the model effectively distinguishing between lower and higher HER2 scores. The comparatively lower AUC for HER2 0, 1+, and 2+ versus class 3+ reflects the challenge of differentiating between HER2 2+ and 3+ scores, which is a known issue in HER2 classification due to the subtle differences in staining intensity. The ROC curves for these tasks are also presented in Fig. S1.

## Discussion

In this study, we introduced a DL-based method that utilizes pyramid sampling to automate the classification of HER2 status in IHC-stained tissue images. By leveraging a hierarchical approach that intricately analyzes features across multiple spatial scales, our method addresses the challenge of HER2 expression heterogeneity, without ROI selection prior to model training. The success of our approach is substantiated through quantitative analysis involving 523 core images from 300 patients never seen before in the training or validation, achieving a classification accuracy of 84.70% compared to the consensus scores obtained from 5 board-certified pathologists. This robust performance underscores not only the method’s precision in captured details but also its potential to mitigate the challenges currently faced in clinical and research settings, such as observer inconsistency and protracted diagnostic timelines.

**Fig. 6.**
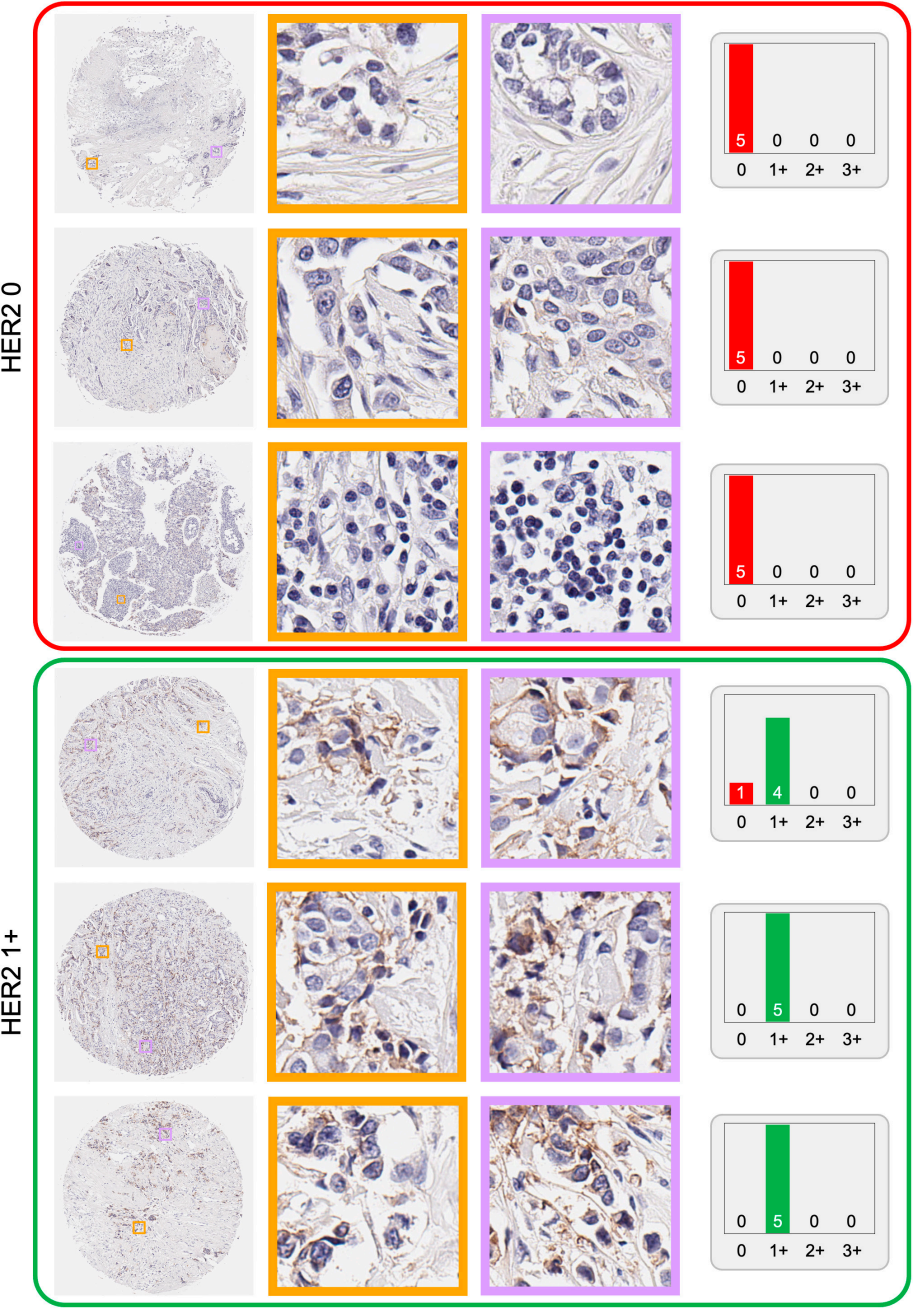
Microscopic examination of HER2 scoring regions in tissue samples. Color-coded boxes display core images from HER2 0 and HER2 1+ tissue sample categories with 2 magnified patches to showcase the specific histological details. Accompanying histograms indicate the predicted score distribution for the highest confidence *PSS*s, offering insights into our automated HER2 assessment.

**Fig. 7.**
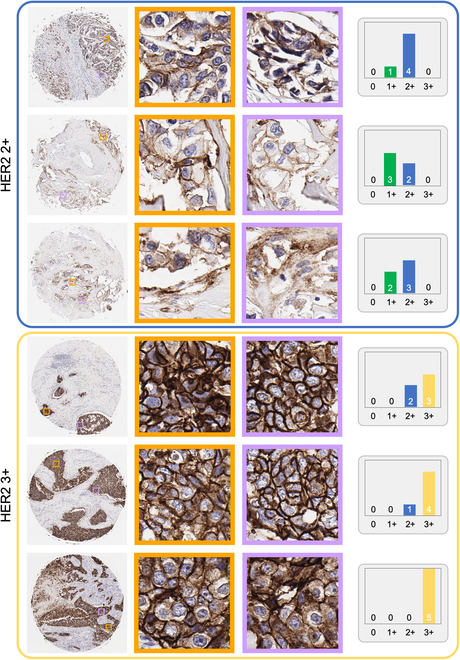
Same as Fig. 6, except for higher HER2 score categories. Color-coded boxes correspond to samples within the HER2 2+ and HER2 3+ tissue sample categories. Two detailed patches are extracted from each core image, emphasizing the staining intensity and cellular patterns critical for determining the respective HER2 scores. Histograms adjacent to each sample reflect the predicted score distribution from the most confident *PSS*s.

The pyramid sampling strategy and inference protocol introduced in our study represent a major advancement in the automated classification of HER2 status, marking, to our knowledge, the first instance of utilizing multi-scale feature analysis for automated HER2 scoring in IHC-stained tissue images. Our pyramid sampling strategy marks a notable departure from previously described methods, focusing on the detailed analysis of both membranous features at the cellular level and broader tissue regions. While most of the previous HER2 classification tools in the literature rely solely on localized patches from a single-scale WSI image [16–18,20–22], we combined high-resolution image patches with their lower-resolution counterparts, ensuring that both the microenvironment of cellular features and the macro-context of tissue architecture are captured and analyzed through our digital framework. This results in a balanced presentation of both the high spatial frequency details and a comprehensive sample field of view in the input of our classification network, which is important to mitigate the HER2 expression heterogeneity observed in tissue samples. This ability of our approach to mitigate the heterogeneity of HER2 expression is also exemplified in Figs. 6 and 7. These figures collectively emphasize the importance of employing multiple *PSS*s covering different spatial scales to navigate the complexities of HER2 scoring, showcasing the system’s adeptness at identifying subtle differences in HER2 biomarker expression levels within the same score category. Furthermore, our method employs a random patch selection mechanism, choosing a specific number of patches at each iteration. This strategy greatly reduces the computational load, enhancing the efficiency of our method without sacrificing the quality and accuracy of tissue characterization.

Beyond the model’s overall accuracy, it is informative to examine its performance in distinguishing between adjacent HER2 classes. The accuracy figures calculated for distinguishing between adjacent HER2 categories demonstrate the model’s effectiveness and are suggestive of its practical utility in a clinical setting. For example, our model achieves an accuracy of 89.62% for differentiating between 0 and 1+ HER2 scores. This is clinically significant because a score of 0 will follow a treatment regimen without using HER2-targeted therapies, while a score of 1+ might prompt further analysis according to recent ASCO/CAP data [7]. A high accuracy in this range minimizes the risk of patients being incorrectly excluded from receiving HER2-targeted treatments if they might benefit from them. For the 1+ versus 2+ categories, the model shows an accuracy of 84.68%. HER2 2+ cases often require additional confirmatory tests such as fluorescence in situ hybridization (FISH) to make a final treatment decision [27,28]. A high accuracy in discriminating HER2 1+ and 2+ helps ensure that patients are appropriately triaged for FISH testing while avoiding unnecessary procedures for others. Finally, the model’s accuracy in distinguishing between 2+ and 3+ scores is 87.26%, ensuring that patients with strong HER2 positivity are promptly identified for appropriate therapeutic intervention.

The practical implications of our DL-based approach for HER2 status classification touch upon 2 pressing issues in pathological assessment: the consistency of manual evaluations and the efficiency of the diagnostic process. Manual evaluation of HER2 IHC status is susceptible to a degree of subjectivity inherent in pathologist judgment. Assessment of data from the CAP surveys demonstrated poor agreement in the evaluation of 0 and 1+ cases (26% concordance) and 58% concordance between 2+ and 3+ [29]. Such vast differences in HER2 quantification can have profound implications on treatment decisions and ultimately patient outcomes. By algorithmically standardizing the scoring process, our method introduces a level of consistency unattainable through manual inspection. This consistency allows our presented model to serve as a valuable enhancement tool for pathologists, offering a consistent second opinion free from human fatigue, level of experience, or bias. This is especially critical in low volume pathology departments that may lack specialized breast pathology experts. In such settings, our automated method can function as a stand-in consultant, providing assessments that can be trusted to align with what a specialist might determine.

The second substantial advantage of our system lies in its potential to markedly shorten the diagnostic turnaround time. Delays in diagnosis can have detrimental effects on patient care, inducing patient dissatisfaction [30], anxiety, and stress [31]. Our approach radically shortens the assessment time to seconds per case, a reduction that can have far-reaching implications in the clinical setting, especially in diagnostically challenging cases. Moreover, in busy pathology departments where the volume of cases can be overwhelming, the efficiency of our method could prevent bottlenecks and reduce interpathologists’ consultations. By allowing for quicker throughput of cases, pathologists can allocate more time to complex cases where human expertise is indispensable.

To provide further insights into the model’s decision-making process, we analyzed the most confident *PSS*s that contributed to the final HER2 score predictions of challenging samples, particularly those near the classification boundary. Figures S2 to S11 illustrate these *PSS*s for 2 sample cores, showing the top 5 most confident *PSS*s for each. In these figures, we observe that the classifier is occasionally “tricked” by certain *PSS*s, resulting in misclassifications. For instance, in the first sample, misclassifications occurred twice (Figs. S2 and S3), and in the second sample, it occurred once (Fig. S7). One key observation from our analysis is the importance of capturing HER2 expression within our *PSS*s. Due to the random nature of the patch sampling, there are areas on the core that our model may not be exposed to. We found that when more patches in the pyramid capture HER2 expression, the model’s predictions improve. This highlights the importance of comprehensive sampling to cover heterogeneous HER2 expression within the tissue. In cases where the patches fail to adequately capture HER2 expression, the likelihood of misclassification increases.

Monte Carlo simulations were essential for addressing the statistical variability introduced by our pyramid sampling strategy and optimizing the parameters *N* and *k*, enabling us to systematically assess and mitigate the effects of randomness on the model’s performance, ensuring a balanced representation of HER2 expression across tissue samples. By identifying an optimal *N*, we made sure that the model could consistently predict HER2 scores despite the variability in tissue representations. Similarly, optimizing *k* allowed us to focus on the most reliable predictions, enhancing the accuracy and reliability of the final HER2 score. The optimal balance for our model’s operational efficiency occurred at *N* = 20, as demonstrated in Fig. 4, highlighting the importance of balancing the number of *PSS*s and model performance to avoid unnecessary computational costs, crucial for clinical use. Beyond *N* = 20, adding more *PSS*s to the inference protocol did not significantly improve accuracy but maintained prediction consistency. Figure 5 shows how the HER2 scoring accuracy decreased when the inference hyperparameter *k* exceeded 20, emphasizing the need to keep *k* within a certain range for optimal classification. This result indicated the delicate balance required between the number of high-confidence predictions and their quality, with a higher *k* value increasing the risk of including less accurate predictions and potentially leading to more false positives.

In the data augmentation process, we did not include color enhancement methods such as color normalization since our entire dataset was acquired using the same slide scanner with limited color variations. On the other hand, we carefully cleaned our dataset before training for better convergence by taking the staining quality, tissue quality, and clinical significance into consideration. Note that during testing, our protocol is inherently robust to staining/imaging quality issues as predictions from problematic areas will receive lower confidence and be excluded for the final prediction. It is also important to note that when applying our scoring framework to images that exhibit stronger variations, such as those acquired from different scanners or under different staining conditions or stained at different laboratories, additional transfer learning may be required for optimal performance. Future work could explore the incorporation of color normalization and other enhancement techniques to improve the model’s robustness and generalizability to external datasets. This would benefit the application of our model to a wider range of clinical settings, ensuring consistent performance across diverse imaging and staining conditions.

In conclusion, our study represents a robust, efficient solution to the challenges of HER2 classification in BC diagnostics. By effectively leveraging pyramid sampling to address the heterogeneity of HER2 expression and demonstrating the potential to streamline diagnostic processes, our approach enhances the accuracy and reliability of HER2 score classification. This research paves the way for more nuanced, faster, and more accessible diagnostics, ultimately contributing to the advancement of personalized medicine and improving patient care in oncology.

## Methods

### Sample preparation, data acquisition, and dataset creation

Fifteen unlabeled breast tissue microarray slides, each containing about 100 to 200 cores, were acquired from TissueArray [32]. These samples underwent HER2 IHC staining at the UCLA Translational Pathology Core Laboratory. We captured bright-field images using a slide scanner microscope (AxioScan Z1, Zeiss) with a ×20/0.8 NA objective lens (Plan-Apo). WSIs were processed to identify and extract each core, utilizing a customized algorithm based on Hough transform techniques [33]. High-resolution bright-field images of the cores were uploaded to Google Photos for pathologist evaluations. To ensure accurate dataset labeling, we employed a voting protocol among 5 board-certified pathologists, where a core’s evaluation needed agreement between at least 2 pathologists. For discordant assessments, an additional pathologist was consulted for a decisive score, leading to the core’s inclusion or exclusion from the dataset. Cores of nondiagnostic quality or those deemed nonscorable were removed. We compiled 2,147 cores with finalized labels, which were allocated into 1,462 for the training set (242 HER2 0, 526 HER2 1+, 314 HER2 2+, 380 HER2 3+), 162 for validation (24 HER2 0, 56 HER2 1+, 35 HER2 2+, 47 HER2 3+), and 523 for blind testing (131 HER2 0, 131 HER2 1+, 126 HER2 2+, 135 HER2 3+).

### Pyramid sampling strategy

We devised a pyramid sampling strategy to capture the multi-scale nature of tissue morphology and HER2 expression patterns. This approach involved systematically extracting small patches from original high-resolution tissue images, approximately ~10,000 × 10,000 pixels in size. We selected forty 512 × 512 pixel patches directly from the original high-resolution image and 10 patches from a 2×-downsampled image, alongside a single patch representing the entire tissue core resized to 512 × 512 pixels. Cropped patches were combined along the channel dimension to create a *PSS*, serving as the input for our DL framework. Figures S2 to S6 present the 5 *PSS*s used in deriving the 5 highest-confidence predictions indicated in the histogram for the topmost tissue core within the green box (HER2 1+) in Fig. 3. Similarly, Figs. S7 to S11 demonstrate the *PSS*s that generated the predictions for the uppermost tissue core within the yellow box (HER2 3+) in the same figure.

The final *PSS* configuration (40 original-resolution patches, 10 2×-downsampled image patches, and 1 resized patch) was selected by hyperparameter tuning on the validation set. As an additional comparative analysis, we optimized the model with 3 other configurations and tested their accuracies on the test set: (a) Obtaining a single patch at the original resolution resulted in a classification accuracy of 73.23%; (b) obtaining 20 patches from the original resolution, 6 from the 2×-downsampled image, and 1 resized core image achieved 79.16% accuracy; and (c) obtaining 20 patches from the original resolution, 6 from the 2×-downsampled image, 2 from the 4×-downsampled image, and 1 resized patch yielded 80.3% accuracy. Our final configuration of 40 original-resolution patches, 10 2×-downsampled image patches, and 1 resized patch provided the best performance. This finding aligns intuitively with the expectation that more patches capture more information, thus enhancing the model’s performance. Additionally, we observed diminishing returns with the addition of another resolution (4×-downsampled image) patches, indicating that further increases in patch numbers and resolution levels do not significantly improve performance while increasing the computational burden. The confusion matrices for these unused hyperparameter cases are included as Fig. S12, which validated the superiority of our selected configuration (see Fig. 5).

### HER2 score classification network architecture and training scheme

We selected DenseNet-201 [34] for our study, a 201-layer densely connected convolutional network, due to its efficiency in image classification. The architecture features dense blocks linked in a feed-forward manner, where each layer receives inputs from all preceding layers and forwards its feature maps to subsequent layers, enhancing feature propagation and reuse.

The network begins with an initial convolutional layer with a kernel size of 7 × 7 × *D_in_* (*D_in_* here represents the number of input channels), followed by batch normalization, a rectified linear unit (ReLU) activation function, and a 3 × 3 max pooling layer with a stride of 2. This is followed by 4 dense blocks [34], each containing a varying number of layers: 6 layers in the first block, 12 layers in the second block, 48 layers in the third block, and 32 layers in the fourth block. Within each dense block, every layer is connected to every other layer. Transition layers between dense blocks include a 1 × 1 convolutional layer followed by a 2 × 2 average pooling layer to manage feature map dimensions. The classification layer was customized to the 4 HER2 score categories (0, 1+, 2+, 3+), employing a softmax activation function to provide the class probability distribution.

To address the class imbalance present in our training set, we implemented a balanced-class weighted cross-entropy loss function. The loss for each class was weighted inversely proportional to its frequency in the training data. This weighting scheme allows the model to focus equally on all classes during the learning process, preventing the dominance of any single class due to its higher occurrence in the dataset. Specifically, for the entire training set, the loss (*L*) for a batch of size (*m*) is given by:

$$L=-\frac{1}{m}\sum_{i=1}^{m}\sum_{c=1}^{C=4}w_{c}\;y_{i,c}\;\text{log}\left(p_{i,c}\right)\;$$

where *C* is the number of classes (4 in our case), *w_c_* represents the class weight, *y*_*i*, *c*_ is a binary indicator of whether class label *c* is the correct classification for observation *i*, and *p*_*i*, *c*_ is the predicted probability of observation *i* for class *c*. Weights *w_c_* help to amplify the signal from underrepresented classes, driving the model to pay more attention to these classes during training. Furthermore, we applied data augmentation techniques, including horizontal and vertical flips and rotations of 90 degrees, to each image in the training set to mitigate overfitting and enhance generalizability of the trained model. The training process of our presented methodology is illustrated in Fig. 2C.

### HER2 score prediction (inference) protocol

Following the generation of *N* distinct predictions for each tissue core, our protocol selects a subset of *k* predictions, *KCS*, based on their confidence levels. This process involves analyzing softmax class probabilities to identify predictions with a clear preference for one HER2 category, indicative of high confidence. The selection criterion focuses on the disparity in HER2 class probabilities within each prediction’s softmax output. The final HER2 score for a tissue core is determined by aggregating the scores within the *KCS*, specifically by taking the maximum score observed across this set. Mathematically, the final HER2 score is given by the maximum score observed across the *KCS*, denoted:

$$\text{Final}\;\text{HER}2\;\text{Score}=\underset{\hat{y}\in \mathrm{KCS}}{\text{max}}\hat{y}$$

where $\hat{y}$ represents the individual HER2 score predictions generated by independent *PSS*s.

Apart from the overall classification accuracy, we also investigated specificity and ROC curves to evaluate the performance of our models. Since this is a 4-class classification, we calculated class-based specificity values. Specificity for each class *i* was derived using the formula:

$${\text{Specificity}}_{i}=\frac{{\text{TN}}_{i}}{{\text{TN}}_{i}+{\text{FP}}_{i}}$$

where *TN_i_* represents the number of instances correctly identified as not belonging to class *i* and *FP_i_* represents the number of instances incorrectly identified as belonging to class *i*. For the ROC curves, we analyzed 3 binary classification tasks: (a) HER2 0 versus HER2 1+, 2+, and 3+, (b) HER2 0 and 1+ versus classes 2+ and 3+, and (c) HER2 0, 1+, and 2+ versus HER2 3+. For each binary task, we binarized the true labels accordingly. Using the softmax probability output by our inference model, we calculated the false positive rates and true positive rates at various threshold settings to plot the ROC curves. The AUC values were computed for each ROC curve, providing a single scalar value to summarize the model’s performance for each binary classification task. For these calculations, we kept *N* = 20 and *k* = 5, averaging the top 5 most confident class probabilities in our calculations.

### Statistical analysis and Monte Carlo simulations

Monte Carlo simulations were utilized to analyze our HER2 score prediction model, focusing on optimizing the number of *PSS*s used in inference (*N*) and refining the selection criteria based on confidence (*k*). These simulations aimed to find an optimal balance that improves accuracy, reliability, and computational efficiency. To evaluate the impact of the inherent variability from our pyramid sampling strategy, 300 independent *PSS*s were generated for each sample in our testing set, providing diverse views of each tissue’s HER2 expression. A range of values for *N*, from 1 to 200 *PSS*s per tissue sample, with *k* fixed at 5, were explored to identify an optimal *N* that enhances the model’s consistency and robustness. Additionally, we investigated the optimal threshold for *k* by varying its values from 1 to 20, and selected higher values [30, 50, 100], with *N* set to a high value of 200, to refine the selection of high-confidence predictions for the final HER2 score determination and to ensure that we could investigate the effects of a wide range of *k* values. These simulations were conducted 10,000 times for each configuration of *N* and *k* to provide a statistically robust analysis.

### Implementation details

The classification network’s training was optimized using the AdamW optimizer [35], an advanced variant of the Adam optimizer designed to incorporate weight decay, thereby mitigating the risk of overfitting. Training commenced with an initial learning rate set at 10^−5^, which was dynamically adjusted in response to changes in the validation loss, with a batch size maintained at 12. This setup allowed the network to reach convergence after approximately 60 h of dedicated training. For a core image resolution of 10,000 × 10,000 pixels, the typical inference time was reduced to less than 15 s, achieved when the inference hyperparameter *N* was set at 20. The training and testing were conducted on a desktop computer equipped with a GeForce RTX 3090 Ti graphics processing unit, 128 GB of random-access memory, and an AMD Ryzen 9 5900X central processing unit. The classification network was implemented using Python version 3.12.0 and PyTorch version 1.9.0, alongside CUDA toolkit version 11.8.

## Data Availability

DL models reported in this work used standard libraries and scripts that are publicly available in PyTorch module. All data supporting the results of this study are available in the main text and the Supplementary Materials.
