# Supplementary material for: Automated HER2 Scoring in Breast Cancer Images Using Deep Learning and Pyramid Sampling
Source: BME Front. 2024 Jul 23;5:0048. doi: 10.34133/bmef.0048 (PMC11265840; doi:10.34133/bmef.0048)
Supplement: Supplementary 1 — Figs. S1 to S12 [file bmef.0048.f1.pdf]

Supplementary Information for Automated HER2 Scoring in Breast Cancer Images Using Deep Learning and Pyramid Sampling

Sahan Yoruc Selcuk[1,2,3], Xilin Yang[1,2,3], Bijie Bai[1,2,3], Yijie Zhang[1,2,3], Yuzhu Li[1,2,3], Musa Aydin[1,2,3], Aras Firat Unal[1,2,3], Aditya Gomatam[1,2,3], Zhen Guo[1,2,3], Darrow Morgan Angus[4], Goren Kolodney[5], Karine Atlan[6], Tal Keidar Haran[6], Nir Pillar[1,2,3] and Aydogan Ozcan[1,2,3,7]

[1]Electrical and Computer Engineering Department, University of California, Los Angeles, CA, USA

[2]Bioengineering Department, University of California, Los Angeles, CA, USA

[3]California NanoSystems Institute, University of California, Los Angeles, CA, USA

[4]Department of Pathology & Laboratory Medicine, University of California, Davis, Sacramento, CA, USA

[5]Bnai-Zion Medical Center, Haifa, Israel

[6]Hadassah Hebrew University Medical Center, Jerusalem, Israel

[7]David Geffen School of Medicine, University of California, Los Angeles, CA, USA

Contents:

Supplementary Figure 1. Receiver operating characteristics (ROC) curves and the area under the curve (AUC) values for the three binary classification tasks.

Supplementary Figure 2. A misclassified PSS that contributed to one five most confident predictions for the primary tissue core in the yellow box of main text Fig. 3, resulting in a HER2 score of 2+.

Supplementary Figure 3. A misclassified PSS that contributed to one five most confident predictions for the primary tissue core in the yellow box of main text Fig. 3, resulting in a HER2 score of 2+.

Supplementary Figure 4. A correctly classified PSS that contributed to one five most confident predictions for the primary tissue core in the yellow box of main text Fig. 3, resulting in a HER2 score of 3+.

Supplementary Figure 5. A correctly classified PSS that contributed to one five most confident predictions for the primary tissue core in the yellow box of main text Fig. 3, resulting in a HER2 score of 3+.

Supplementary Figure 6. A correctly classified PSS that contributed to one five most confident predictions for the primary tissue core in the yellow box of main text Fig. 3, resulting in a HER2 score of 3+.

Supplementary Figure 7. A misclassified PSS that contributed to one five most confident predictions for the primary tissue core in the green box of main text Fig. 3, resulting in a HER2 score of 0.

Supplementary Figure 8. A correctly classified PSS that contributed to one five most confident predictions for the primary tissue core in the green box of main text Fig 3, resulting in a HER2 score of 1+.

Supplementary Figure 9. A correctly classified PSS that contributed to one five most confident predictions for the primary tissue core in the green box of main text Fig 3, resulting in a HER2 score of 1+.

Supplementary Figure 10. A correctly classified PSS that contributed to one five most confident predictions for the primary tissue core in the green box of main text Fig 3, resulting in a HER2 score of 1+.

Supplementary Figure 11. A correctly classified PSS that contributed to one five most confident predictions for the primary tissue core in the green box of main text Fig 3, resulting in a HER2 score of 1+.

Supplementary Figure 12. Confusion matrices for different sampling configurations.

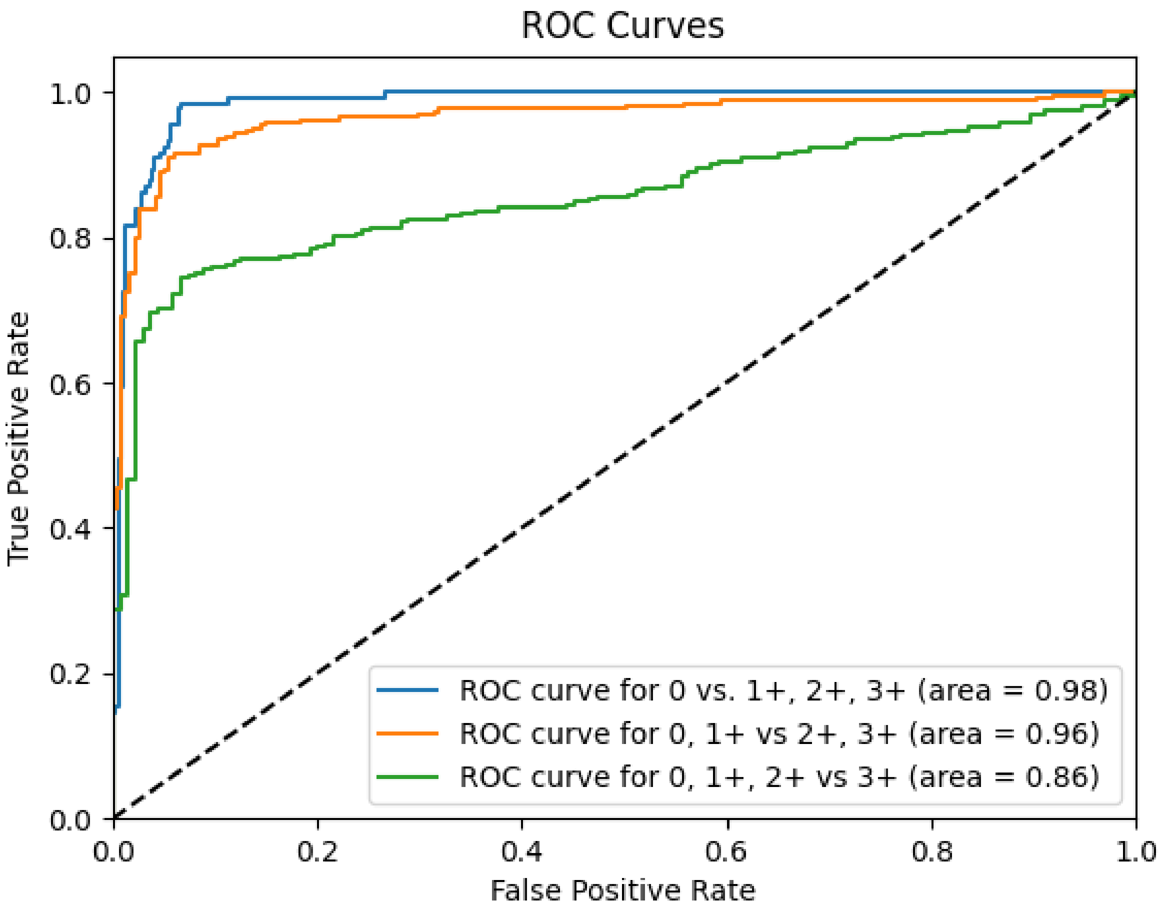

Supplementary Figure 1. Receiver operating characteristics (ROC) curves and the area under the curve (AUC) values for the three binary classification tasks.

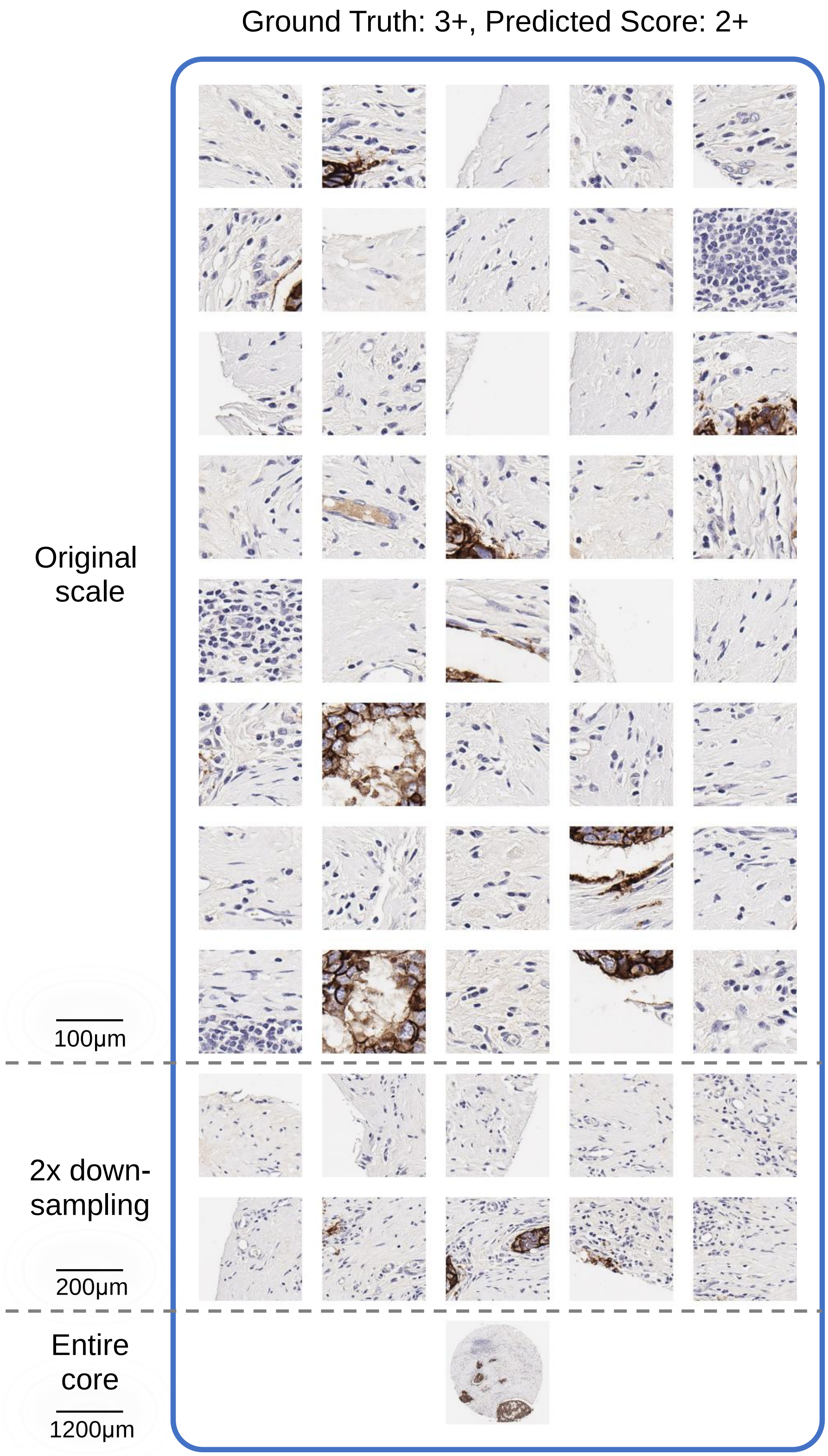

Supplementary Figure 2. A misclassified PSS that contributed to one five most confident predictions for the primary tissue core in the yellow box of main text Fig. 3, resulting in a HER2 score of 2+.

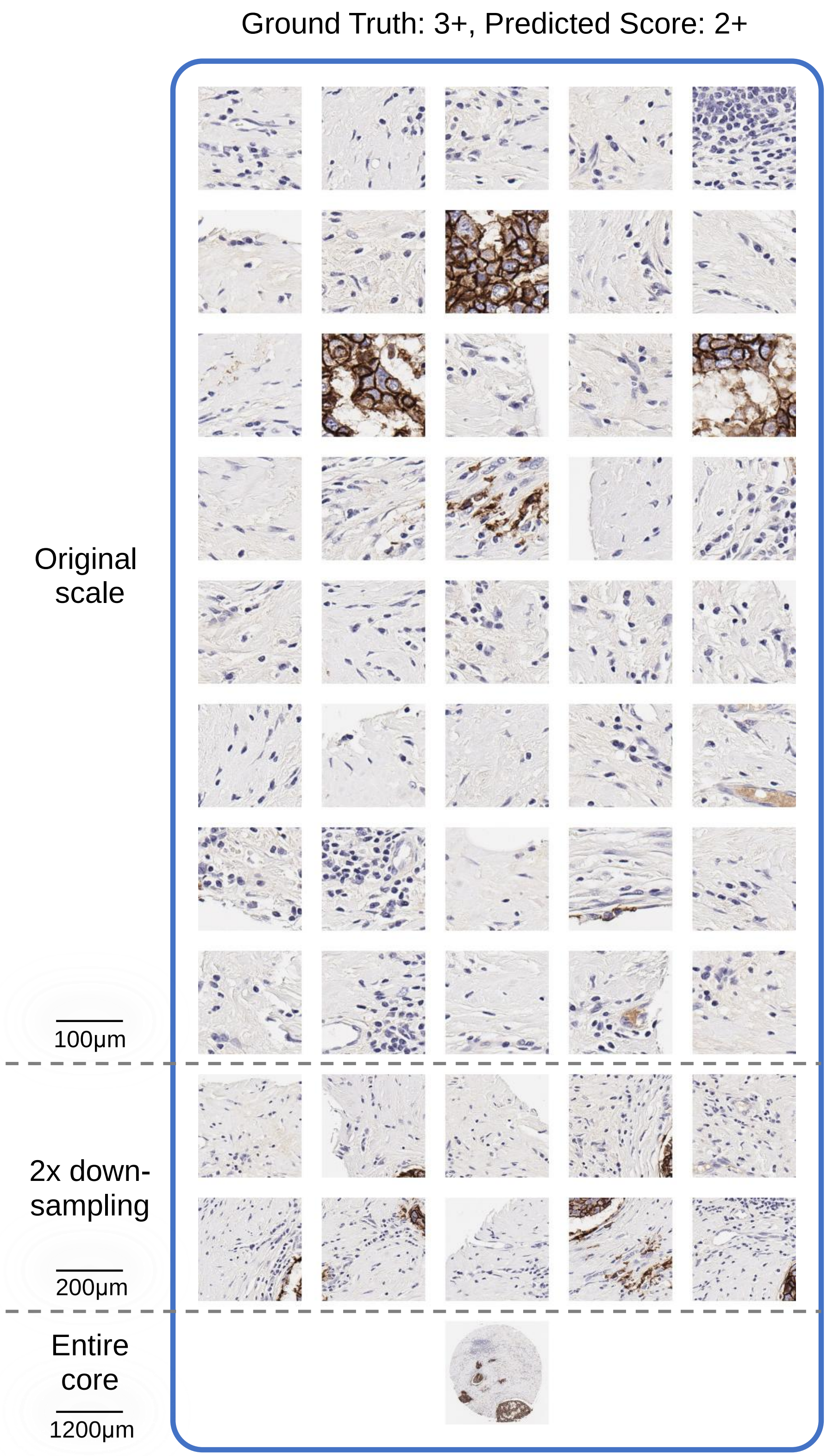

Supplementary Figure 3. A misclassified PSS that contributed to one five most confident predictions for the primary tissue core in the yellow box of main text Fig. 3, resulting in a HER2 score of 2+.

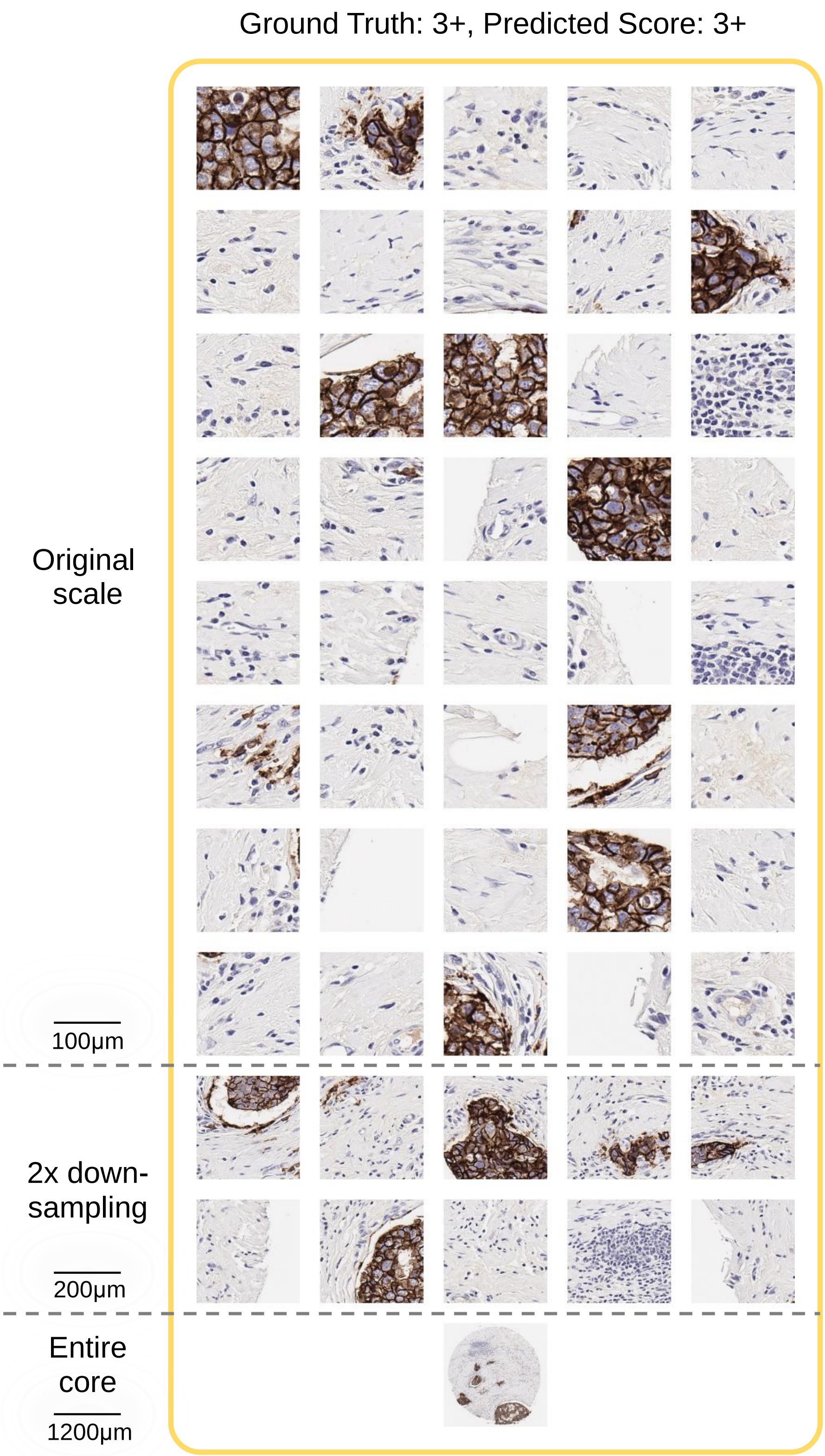

Supplementary Figure 4. A correctly classified PSS that contributed to one five most confident predictions for the primary tissue core in the yellow box of main text Fig. 3, resulting in a HER2 score of 3+.

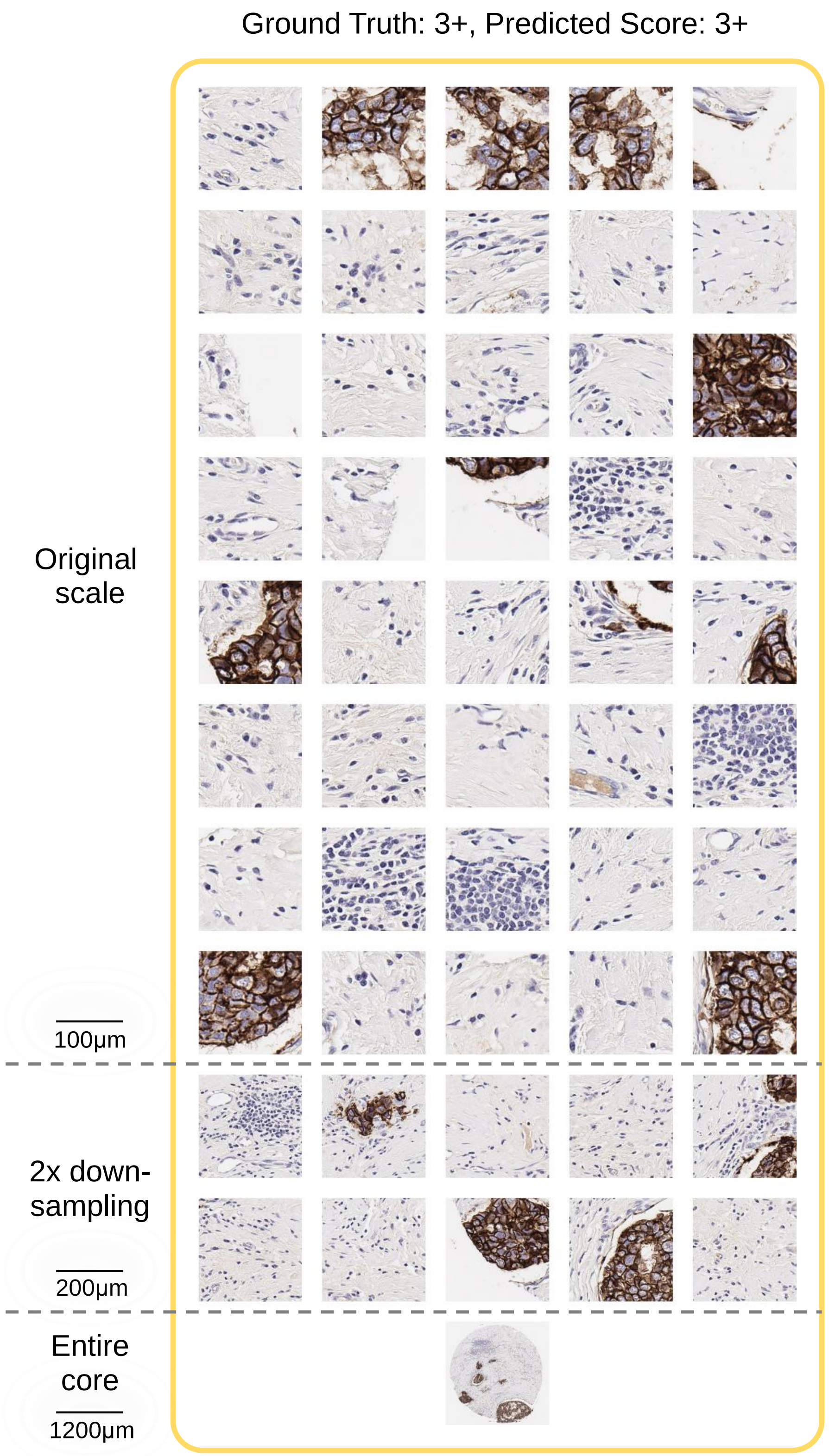

Supplementary Figure 5. A correctly classified PSS that contributed to one five most confident predictions for the primary tissue core in the yellow box of main text Fig. 3, resulting in a HER2 score of 3+.

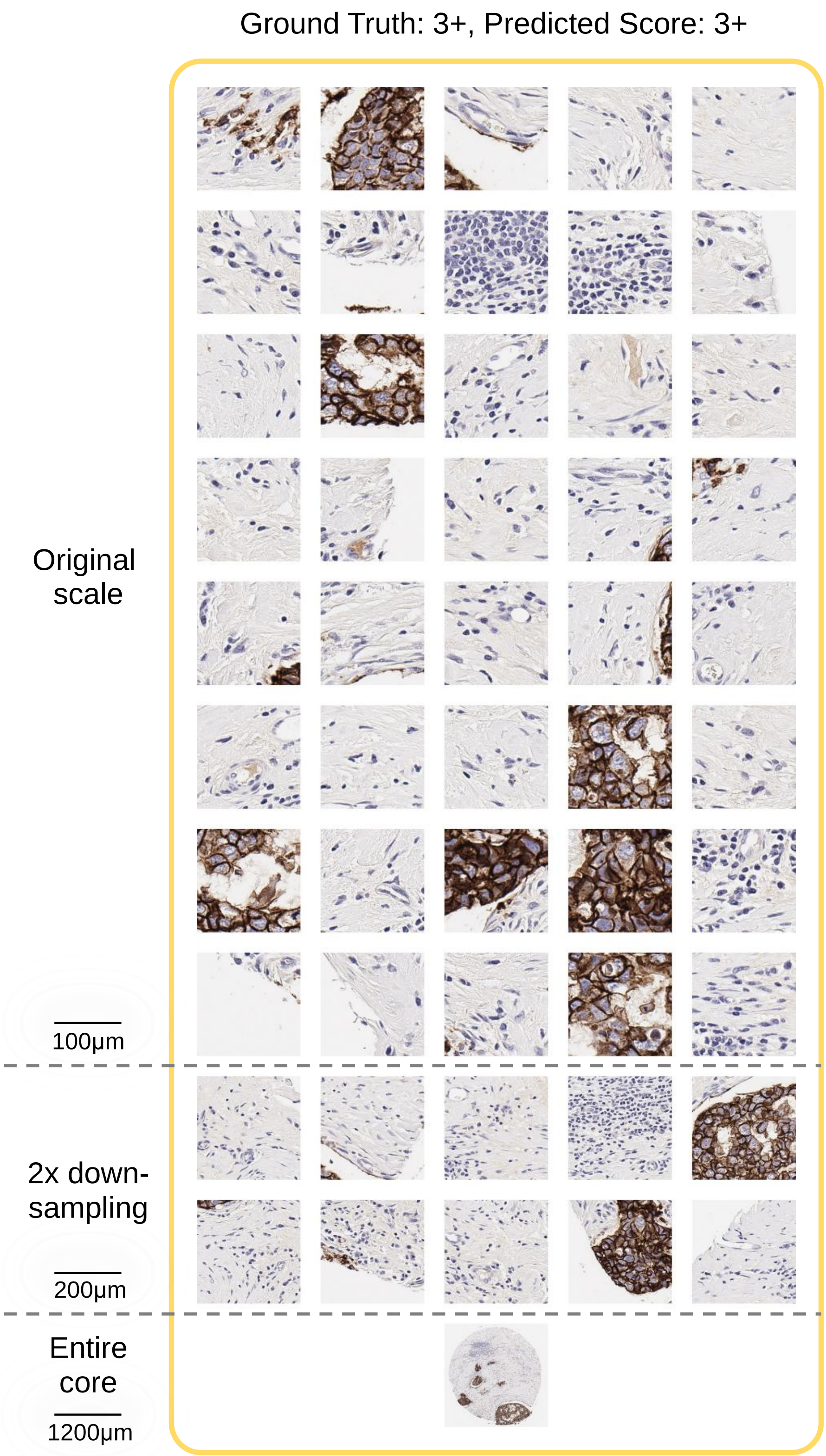

Supplementary Figure 6. A correctly classified PSS that contributed to one five most confident predictions for the primary tissue core in the yellow box of main text Fig. 3, resulting in a HER2 score of 3+.

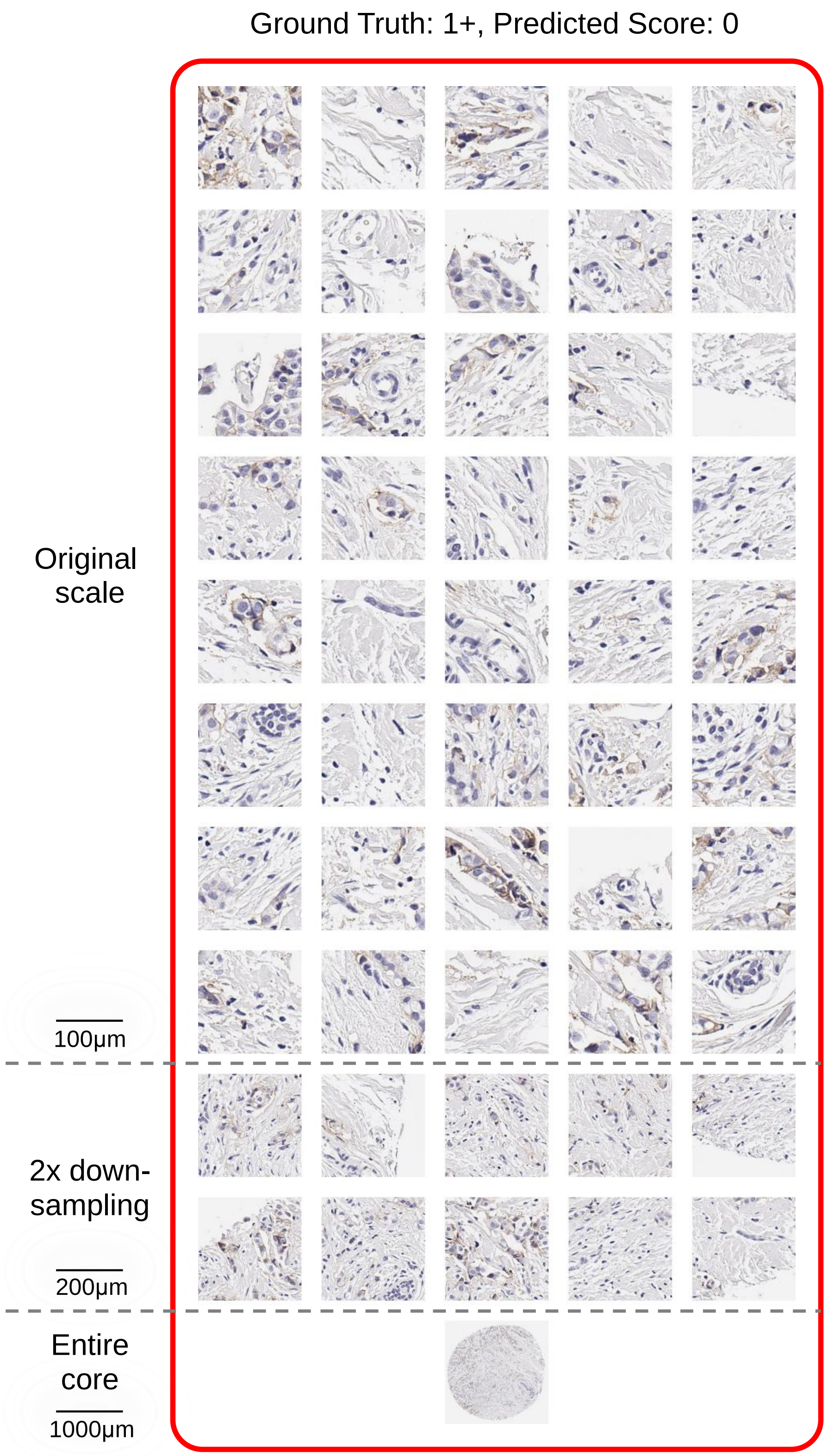

Supplementary Figure 7. A misclassified PSS that contributed to one five most confident predictions for the primary tissue core in the green box of main text Fig. 3, resulting in a HER2 score of 0.

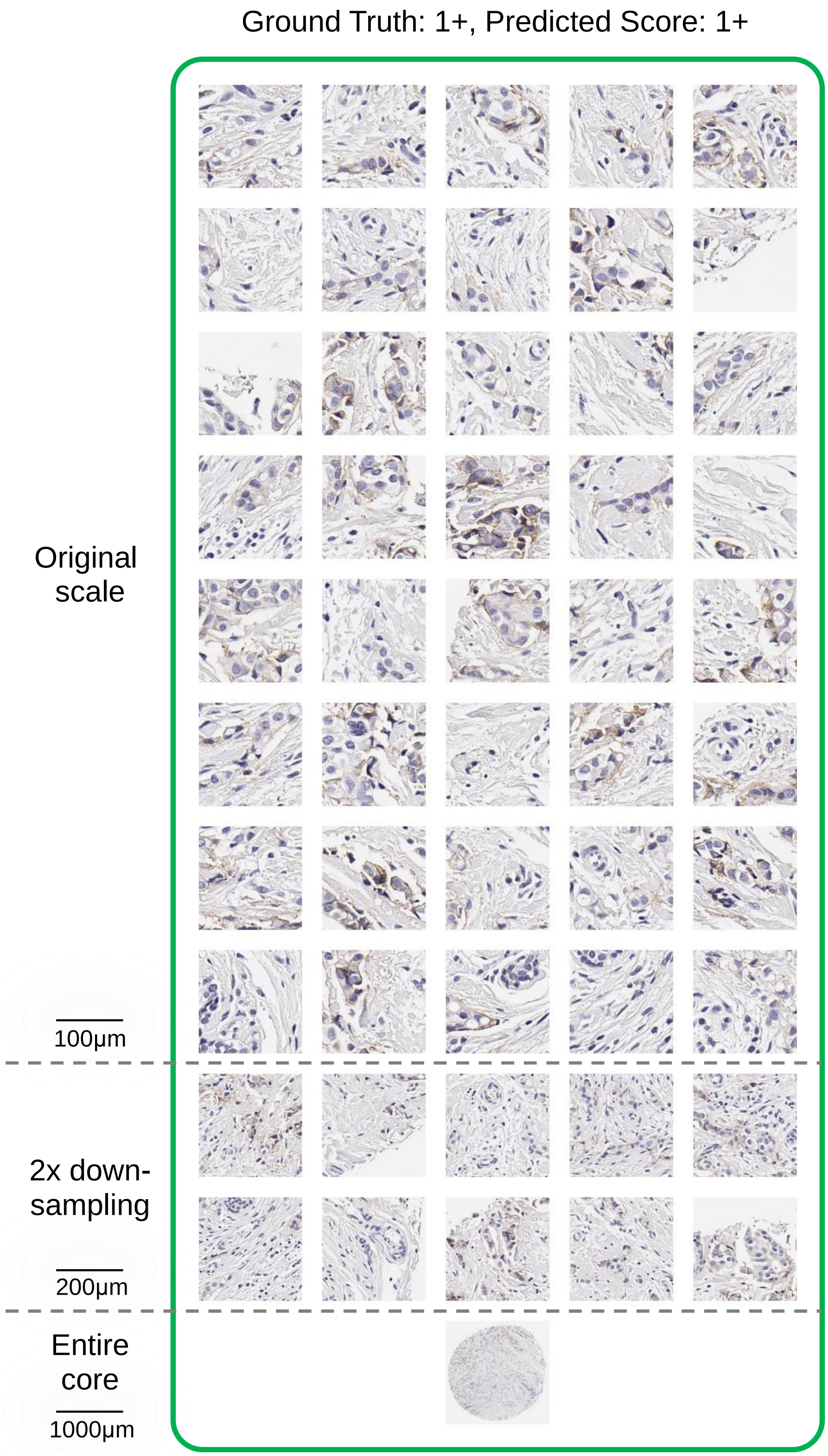

Supplementary Figure 8. A correctly classified PSS that contributed to one five most confident predictions for the primary tissue core in the green box of main text Fig 3, resulting in a HER2 score of 1+.

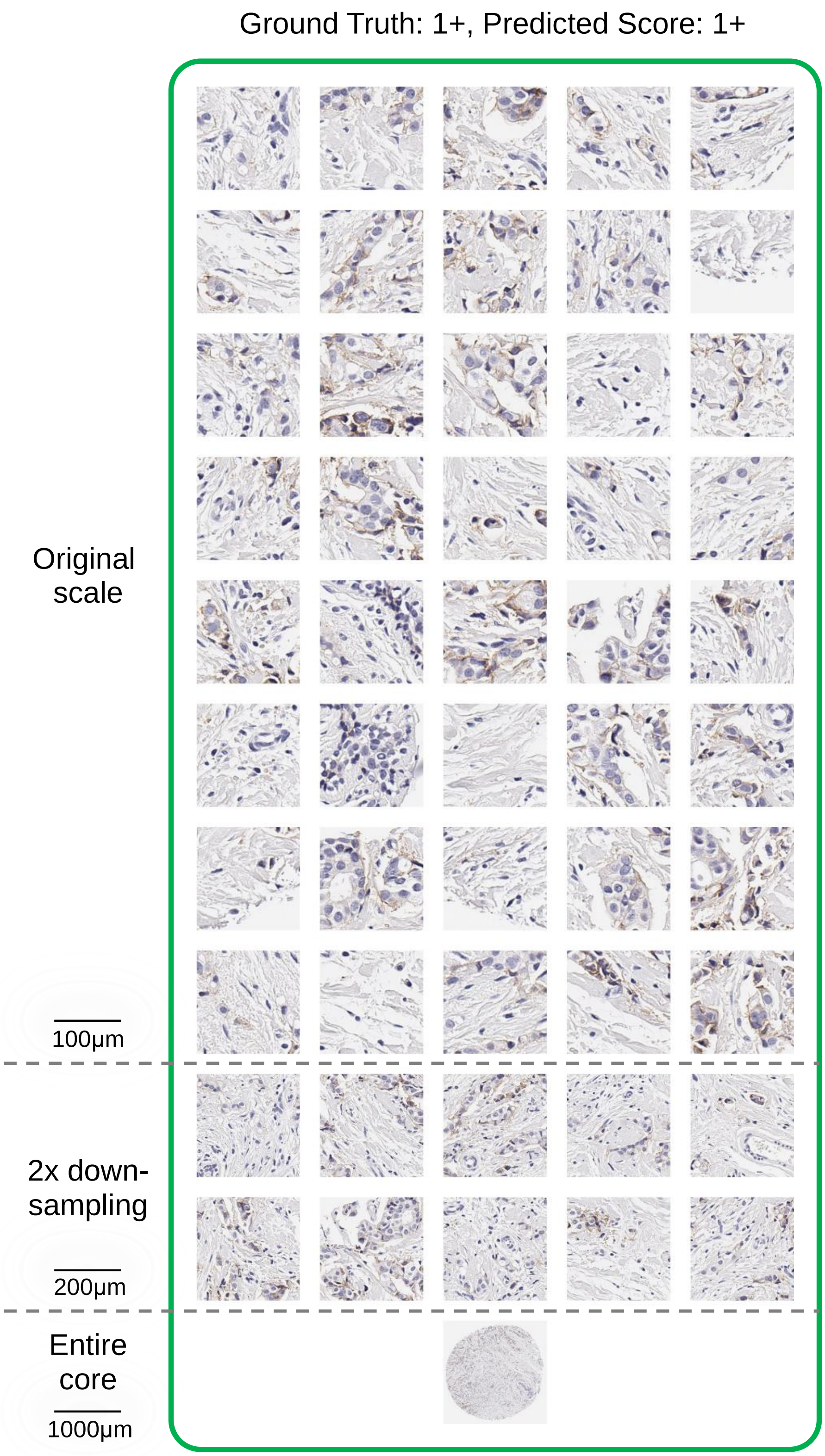

Supplementary Figure 9. A correctly classified PSS that contributed to one five most confident predictions for the primary tissue core in the green box of main text Fig 3, resulting in a HER2 score of 1+.

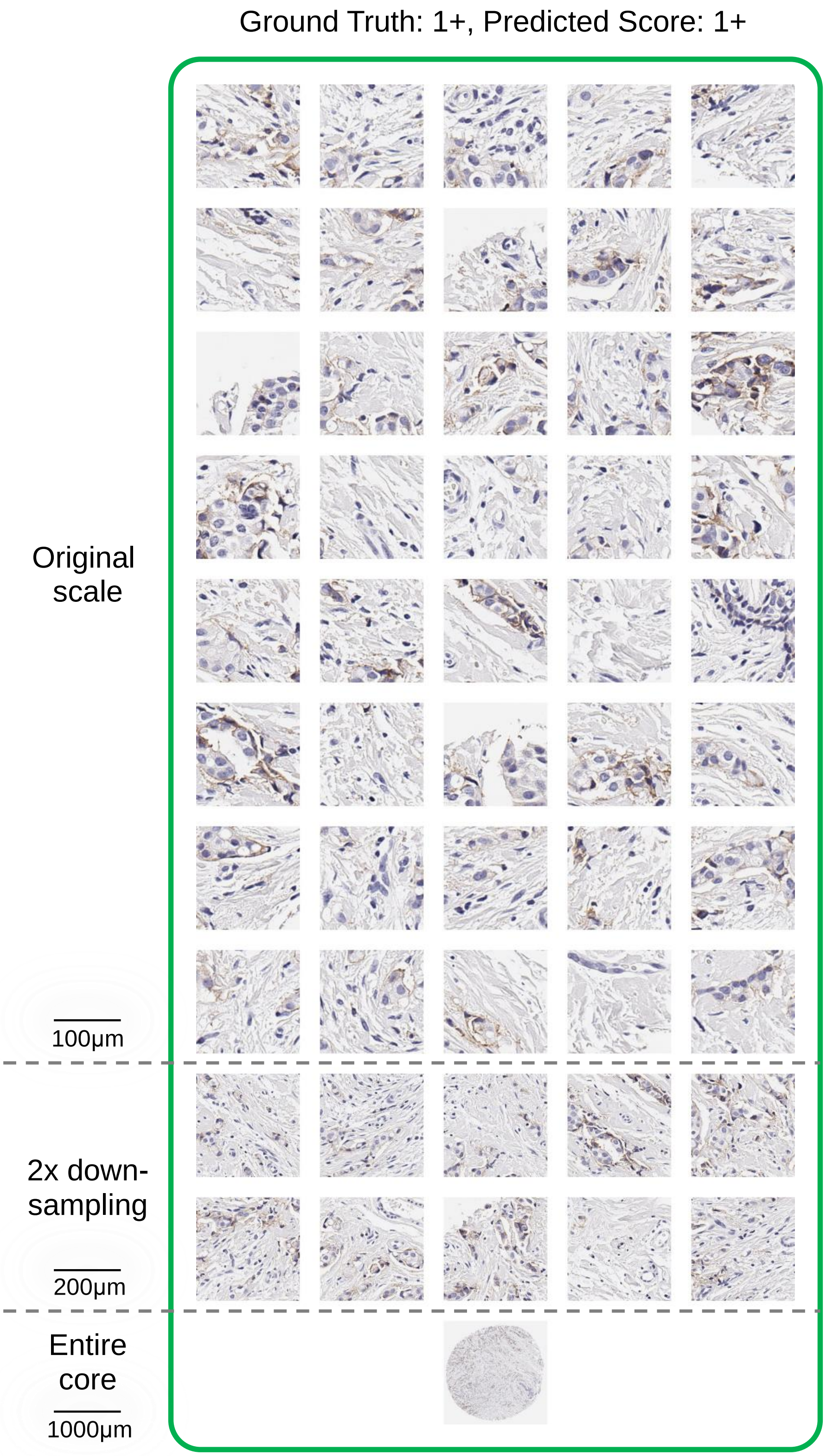

Supplementary Figure 10. A correctly classified PSS that contributed to one five most confident predictions for the primary tissue core in the green box of main text Fig 3, resulting in a HER2 score of 1+.

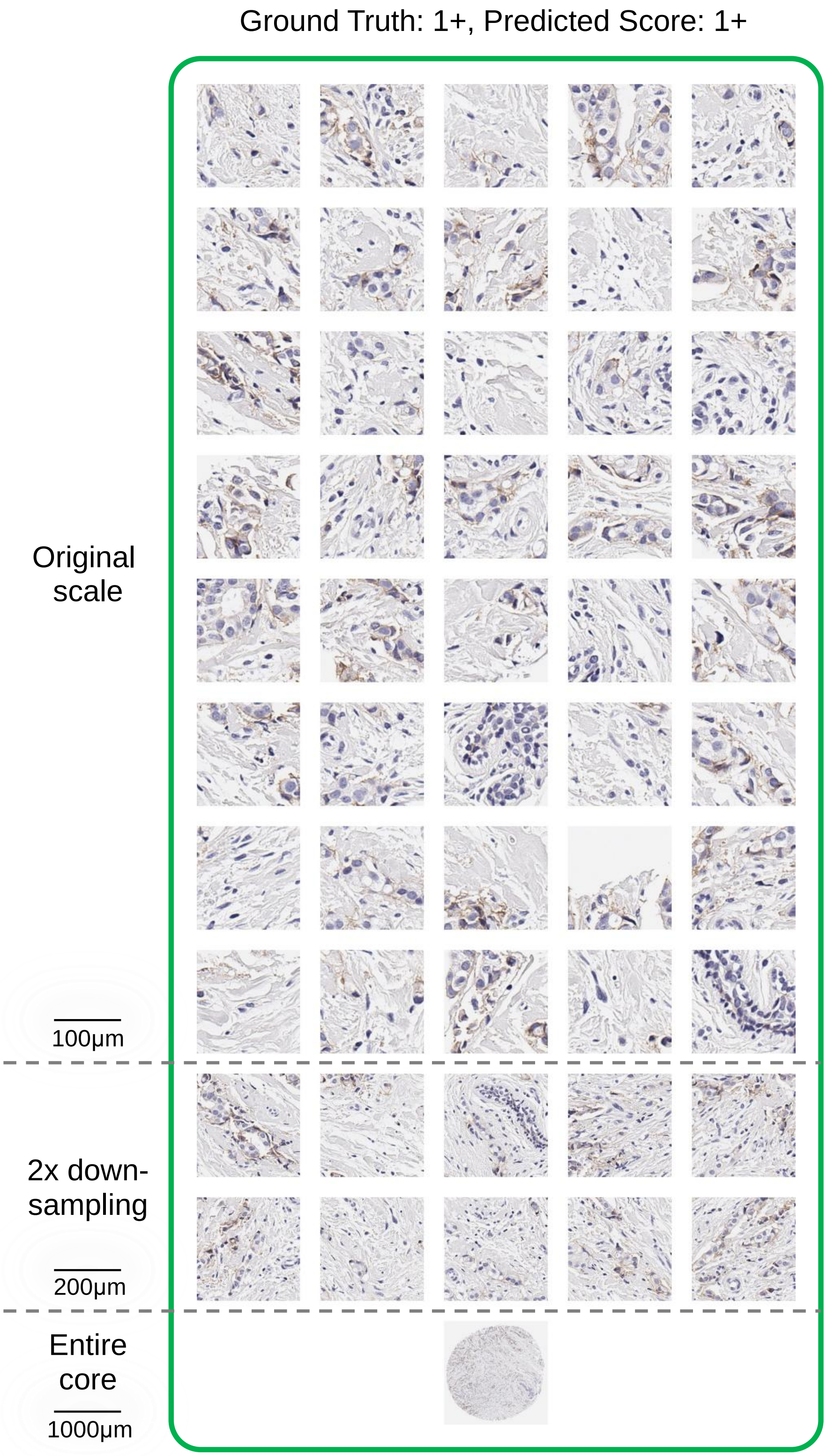

Supplementary Figure 11. A correctly classified PSS that contributed to one five most confident predictions for the primary tissue core in the green box of main text Fig 3, resulting in a HER2 score of 1+.

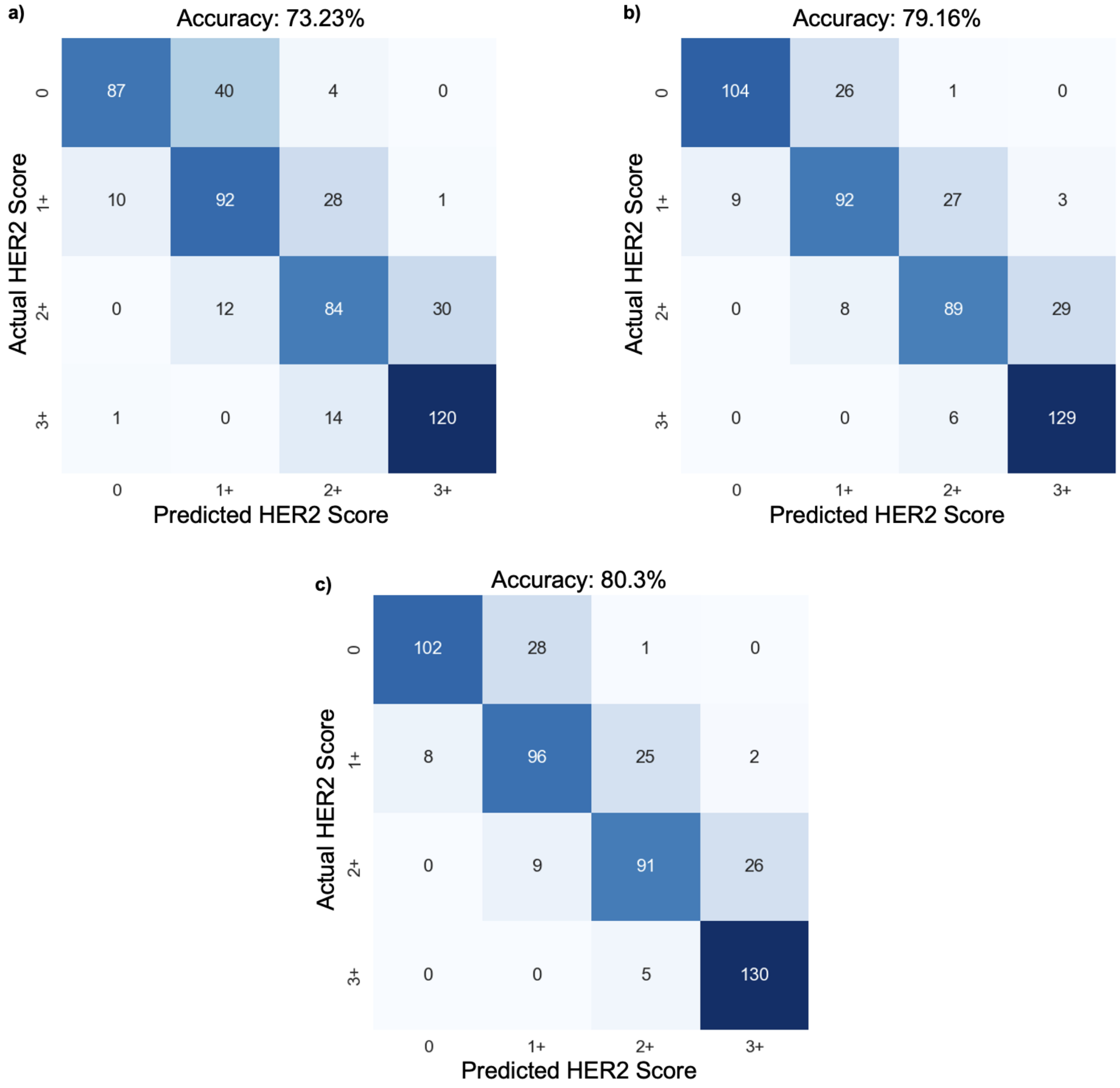

Supplementary Figure 12. Confusion matrices for different sampling configurations. a) Confusion matrix with 1 patch at the original resolution. **b)** Confusion matrix with 20 patches from the original resolution, 6 from the 2x-downsampled image, and 1 resized patch. **c)** Confusion matrix for the sampling configuration with 20 patches from the original resolution, 6 from the 2x-downsampled image, 2 from the 4x-downsampled image, and 1 resized patch.
